# Incidence trend of breast Cancer in women of eastern Mediterranean region countries from 1998 to 2019: A systematic review and meta-analysis

**DOI:** 10.1186/s12905-020-00903-z

**Published:** 2020-03-17

**Authors:** Razieh Zahedi, Hossein Molavi Vardanjani, Mohammad Reza Baneshi, Ali Akbar Haghdoost, Reza Malekpour Afshar, Roghayeh Ershad Sarabi, Fatemeh Tavakoli, Farzaneh Zolala

**Affiliations:** 1 Modeling in Health Research Center, Kerman University of Medical Sciences, Kerman, Iran; 2 MPH Program, School of Medicine, Shiraz University of Medical Sciences, Shiraz, Iran; 3 Pathology and Stem Cell Research Center, Kerman University of Medical Sciences, Kerman, Iran; 4 Medical Informatics Research Center, Institute for Futures Studies in Health, Kerman University of Medical Sciences, Kerman, Iran; 5 HIV/STI Surveillance Research Center, and WHO Collaborating Center for HIV Surveillance, Institute for Futures Studies in Health, Kerman University of Medical Sciences, Kerman, Iran; 6 Social Determinants of Health Research Center, Institute for Futures Studies in Health, University of Medical Sciences, Kerman, Iran

**Keywords:** Breast cancer, Meta-analysis, Eastern Mediterranean region, Incidence

## Abstract

**Background:**

This study was conducted to provide evidence on the current status of breast cancer and its incidence trend in Eastern Mediterranean Region during 1998–2019. Also, this study aimed to investigate the association between the incidence of breast cancer and Human Development Index and some factors related to this index, including total fertility rate, and obesity, using a meta-analysis.

**Method:**

Data on incidence of breast cancer were collected from various sources, including PubMed, Embase, Web of Science, and WHO, from 1998 to 2019 using systematic review and meta-analysis. Pooled age standardized rate was calculated based on study duration and quality of data using a subgroup analysis and random effect meta-analysis.

**Results:**

A total of 80 studies (545 data points) were analyzed. Pooled age standardized rate of breast cancer for Eastern Mediterranean Region was 37.1 per 100,000 person-year (95% confidence interval [CI], 34.5, 39.8) during 2011–2019. age standardized rate of breast cancer had an upward trend in Eastern Mediterranean Region from 2005 to 2019. However, the increasing trend was found to be slightly different in various regions based on quality of data. Moreover, pooled age standardized rate had a significant association with Human Development Index [− 89.2 (95% CI, − 119.8, − 58.7)] and obesity [1.2 (95% CI, 0.9, 1.5)].

**Conclusion:**

Pooled age standardized rate of breast cancer in Eastern Mediterranean Region was lower than the global average. Also, the age standardized rate value and its incremental trend have been higher in countries with high-quality data than in other countries of this region in recent years. Data quality or physiological factors, such as increase in obesity rates, could be the reasons for this incremental trend.

## Background

Breast cancer (BC) is the most prevalent cancer among women worldwide and has also been the fifth leading cause of death among cancers in both sexes globally between 2005 and 2015 [1]. According to previous studies, BC will be one of the most important causes of death in women in reproductive age in developing countries in the future [2]. The average age at presentation of BC was less in Asian women compared to those in Western countries [3–7]. previous studies showed the age standardized rate (ASR) of BC in women increased from 58 to 65.5 per 100,000 person-year worldwide during 2005–2015. A more incremental trend of ASR in BC has been observed in countries with low socioeconomic index than in countries with a high socioeconomic index. In 2015, the lowest ASR of BC belonged to Southeast Asian countries, with 35.8 (95% CI, 27.5–45.4), followed by South Asian countries, with 44.4 per 100,000 person-year (95% CI, 37.1–52.3) [1]. According to GLOBOCAN 2018, the 5-year prevalence of BC was 88.8 per 100,000 women in Eastern Mediterranean Region (EMR) [8].

According to World Health Organization (WHO), EMR comprises of 21 countries, with the population of about 583 million. The countries in this region have diverse economic, social, health indexes, and life expectancy [9].

In some studies, socioeconomic factors have been mentioned as possible factors influencing BC incidence [1, 10]. Human Development Index (HDI), a composite index comprised of life expectancy at birth, salary, and education, is one of the indicators used to study the level of welfare in human societies [11]. Considering the lack of proper cancer registration systems and surveillance structure in most of developing countries, particularly in EMR countries [12], and given that EMR includes countries with diverse socioeconomic and health status [9], similar patterns may be found in this region and in other similar countries in the present and future. This study was conducted to provide evidence on the current status of BC and its incidence trend in the EMR during1998–2019. Moreover, this study investigated the association between the incidence of BC and HDI and some factors related to this index, including total fertility rate (TFR) and obesity, using a meta-analysis.

## Methods

### Search strategy

Studies were selected using a systematic and comprehensive search of the literature, review of references, government publications, and recommendations by active researchers in the field. Electronic databases were searched using the following keywords: “breast neoplasms”, “breast cancer”, “breast tumor”, “incidence”, “frequency”, “distribution”, and “epidemiology” by adding the names of all the 21 EMR countries separately (Web table1). Relevant studies were identified by searching the WHO Global Index Medicus Database, Medline, PubMed, Embase, and Web of Science. The *grey literature* was found on the websites of WHO, IARC, IRCT, Pecos, Google, and Google Scholar. The papers and reports published up to November 2019 were searched.

### Inclusion and exclusion criteria

All national and international studies and reports on the incidence of BC in EMR countries (Iran, Afghanistan, Pakistan, Qatar, Kuwait, Egypt, Lebanon, Oman, Jordan, Yemen, Iraq, Bahrain, Libya, Morocco, Saudi Arabia, Sudan, Syria, Tunisia, Djibouti, United Arab Emirates (UAE), and Somalia; based on WHO classification) during 1998–2019 were included in this study.

Review studies and abstract papers of conferences and congresses that did not have full-texts and studies conducted on specific age groups or on a specific histology of BC were excluded. When studies with similar data were found, the one with higher quality was selected. Quality assessment of studies was done using JBI (The Joanna Brigge Institute) checklist [13] and risk of bias assessment checklist [14]. Each checklist has 10 items, with the scores ranging from 0 to 10. The items included representativeness of the target population, recruitment methods, adequacy of sample size, reporting details of study methods, appropriateness of measurements, and analysis and reporting methods. These 2 checklists were filled up by RZ&FT independently to decrease risk of bias. Moreover, the validity and reliability of quality assessment checklists between 2 investigators were checked through coefficient agreement. The average scores were calculated using the checklists, and studies with a score below 5 were excluded from the study, based on the average score of both checklists.

### Extracted data

Extracted data included title of the study, DOI (digital object identifier) of the paper, writer’s name, year of the study, location of the study, name of the journal, year of publication, and writer’s address. Specific information included study period, sampling method, sample size, number of BC cases, data collection source, and study results. Also, crude incidence rate and ASR of BC were standardized based on WHO population and standard error (SE), standard deviation (SD), or confidence interval (CI) of these indexes.

### Data manipulation

Crude incidence rate and sample size were used to calculate the number of BC cases when their number was not reported. However, when the number was reported but no information was provided on dispersion index (SD, SE, or CI), Keyfitz formula was used to estimate SE [15]. When neither the dispersion index (such as SE) nor the number of BC cases was reported, SE was obtained using MICE (Multiple Imputation via Chained Equations) [16]. SE was calculated using this method in approximately 13% of the cases.

In cases where a study was conducted during several years and the annual ASR was not reported, but the average of ASR was reported for that period, the mid-point year of the study was considered as a data point. Quality assessment of the studies and data extraction were done by 2 investigators (ZR & TF) independently. Disagreement between the investigators was resolved by discussion and review or by referring to the third author (ZF).

### Final data used in the analysis

In addition to the data extracted from the systematic review of the literature, other information, such as TFR, was extracted from HDI of United Nations Development Program during 1998–2018 (UNDP) [11] and from Index Mundi website, from which data on the prevalence of obesity in every EMR country were extracted [17].

### Data analysis

All ASR index analyses were run based on SQRT (square root) and according to Poisson distribution due to the positive skewness of the studied variables [18]. Considering the high values of I square index (68.4%, *p* value < 0.001) and Tau^2^ = 2.2(Tau^2^ is the estimated standard deviation of underlying effects across studies), and according to the results of the initial meta-regression analyses, the variables of time, location, and quality of cancer registry system were significant. Therefore, subgroup analysis was done to reduce heterogeneity based on 4 separate time periods (1998–2000; 2000–2005; 2006–2010; and 2011–2019) and also based on 3 categories of the quality of cancer registry system. As the major part of data were reported based on the data of the cancer registry system, the extracted data were categorized according to the quality of cancer registry system in each country. of data, Group 1 had high-quality data and included countries in which the coverage of population-based registry of cancer was over 50% (Qatar, Kuwait, Bahrain, and Oman); group 2 had medium-quality data and included countries in which the coverage of population-based registry of cancer was lower than 50% (Iran, Morocco, Saudi Arabia, Libya, Tunisia, Egypt, and Jordan); and group 3 had low-quality data based on pathology, treatment centers, or survey (Afghanistan, Pakistan, Iraq, Yemen, Djibouti, Somalia, Syria, Sudan, UAE, Lebanon).

In this study, a meta-analysis was done in 3 parts:

#### Determining the current status of breast Cancer

The data on the ASR of BC during 2011–2019 were used to determine the current status of BC. The pooled ASR of BC was calculated separately for each country in EMR using random-effect meta-analysis method.

#### Studying the ASR trend of breast Cancer

To find the trend of BC, data were analyzed based on the data of the whole region from 1998 to 2019 and then based on the quality of data in the corresponding period. Data were analyzed using metan command in random-effects meta-analysis and fractional polynomial regression method for the subgroups based on quality of data.

#### Studying the association between related factors influencing the ASR of breast Cancer

The association between the ASR of BC and HDI, TFR, and obesity were investigated using unadjusted (univariate) and adjusted (multivariate) meta-regression method. In addition, meta-regression method was used to investigate the effect of time, location, and quality of cancer registry system on the heterogeneity of the extracted ASR of BC.

As about 60% of data points were related to Iran, sensitivity analysis was used to estimate the pooled ASR of BC in EMR. In the sensitivity analysis, the results were reported by including the studies related to Iran and then by excluding them. All the analyses were done using STATA 12.0 software (StataCorp, College Station, TX, USA). All *p* values were 2-sided, and significance level was set at less than 0.05.

## Results

**Fig. 1:**
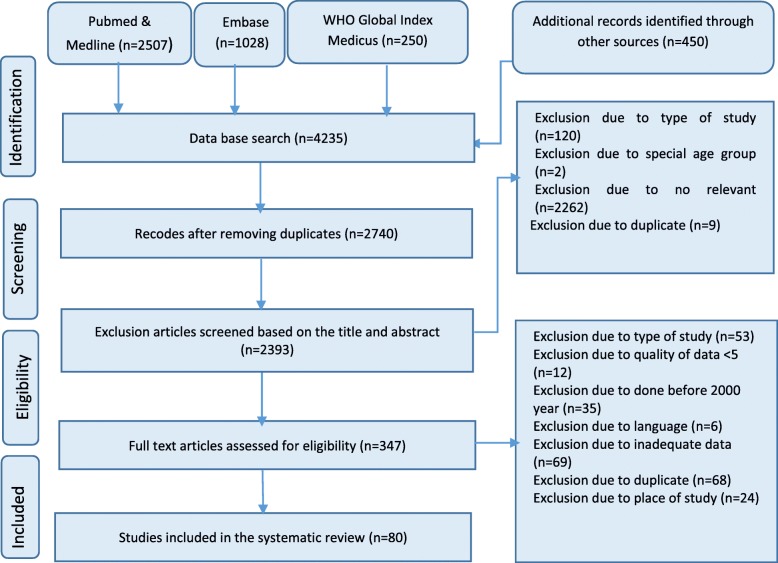
The selection process of papers relevent to the systematic review

In the initial search, 4235 papers, international, and national reports were identified, among which 80 studies met the criteria to be included in the analysis [1, 6, 8, 12, 19–94]. The search processes are illustrated in Fig. 1, and the summarized characteristics of the included studies are presented in supplementary file (Table S2). The average agreement between the 2 researchers (ZR&TF) to select the studies, conduct quality assessment, and data extraction was good (Cohen’s unweighted κ = 0.87). A total of 545 data points of BC incidence were extracted from 80 studies, among which 55% (298 data points) belonged to Iran. In this study, most of the data (77%) belonged to the national cancer registry system and 40% to the third period of the study (2006–2010). Also, 72% of the data belonged to those EMR countries that had medium-quality of data (group 2) (Table 1).

**Table 1:** Characteristics of 544 data points on Adjusted Incidence Rate of breast cancer in Eastern Mediterranean countries, published between 1998 and 2019

Source of Data point	Number of Data point (%)
International report (GLOBOCAN, IARC, Global burden)	103 (18.9)
Cancer registry report (National)	419 (77.02)
Survey, pathology or hospital report	22 (4.04)
Period of study	
1998–2000	42 (7.7)
2001–2005	142 (26.1)
2006–2010	219 (40.3)
2011–2018	141 (25.9)
Quality of Data	
Group1: High Quality (population base coverage over 50%)	63 (11.6)
Group2: Medium Quality (population base coverage lower 10%)	393 (72.2)
Group3: Low Quality of National data (Survey, pathology or hospital report)	88 (16.2)

### The current status of ASR of breast Cancer in countries in EMR from 2011 to 2019

Pooled ASR of BC in EMR was about 37.1 per 100,000 person-year (95% CI, 34.5, 39.8) during 2011–2019. However, after removing the data from Iran, the estimated number was 39.7 per 100,000 person-year (95% CI, 36.03, 42.3); this difference was statistically significant (*p* value < 0.001) (Table 2).

**Table 2:** The ASR of Breast Cancer in Eastern Mediterranean Countries in 2011–2019

Country	Rank for ASR of female breast cancer	HDI index	Quality of Data^a^	N Data point	ASR of breast cancer (95%CI)	Number of breast cancer cases (% of total cases)	Total of sample size (%of total sample size)
Lebanon	1	0.763	3	5	68.9 (50.4, 90.3)	5908 (1.5)	7,717,060 (0.6)
Kuwait	2	0.8	1	4	54.8 (34.8, 79.2)	1396 (0.4)	4,010,811 (0.3)
UAE	3	0.84	3	4	53.3 (34.8, 73.9)	2616 (0.7)	7,357,716 (0.6)
Pakistan	4	0.55	3	8	51.8 (37.2, 68.9)	173,768 (43.9)	281,000,438 (21.9)
Qatar	5	0.856	1	4	49.1 (26.01, 79.02)	9813 (2.5)	26,686,518 (2.1)
Bahrain	6	0.824	1	4	46.2 (24.01, 73.9)	695 (0.2)	1,537,277 (0.1)
Jordan	7	0.742	2	7	44.9 (33.6, 57.8)	5461 (1.4)	16,901,905 (1.3)
Libya	8	0.716	2	6	44.9 (28.1, 64.8)	2714 (0.7)	12,806,644 (0.9)
Djibouti	9	0.473	3	4	40.9 (19.4, 68.9)	598 (0.1)	1,621,024 (0.1)
Iraq	10	0.649	3	5	39.7 (28.1, 53.3)	22,766 (5.7)	51,396,093 (4.0)
Yemen	11	0.482	3	5	37.2 (28.1, 47.6)	448 (0.1)	1,383,852 (0.1)
Sudan	12	0.49	3	5	36.7 (29.2, 44.9)	14,788 (3.7)	56,335,878 (4.4)
Morocco	13	0.647	2	5	36.1 (28.1, 44.9)	25,575 (6.5)	50,864,911 (3.9)
Egypt	14	0.691	2	5	33.6 (26.01, 43.6)	50,002 (12.6)	93,279,217 (7.3)
Afghanistan	15	0.479	3	5	33.6 (26.01, 43.6)	6746 (1.7)	48,588,869 (3.8)
Somalia	16	___	3	3	33.6 (20.2, 49.3)	3689 (0.9)	18,502,099 (1.4)
Tunisia	17	0.725	2	5	32.5 (24.01, 42.3)	7772 (1.9)	16,625,705 (1.3)
Oman	18	0.796	1	7	32.5 (18.5, 51.8)	1191 (0.3)	30,466,497 (2.4)
Iran	19	0.774	2	41	31.9 (28.2, 35.8)	45,641 (11.5)	484,392,544 (37.7)
Saudi Arabia	20	0.847	2	5	30.3 (22.1, 40.9)	6915 (1.7)	39,440,443 (3.1)
Syrian Arab republic	21	0.536	3	5	24.01 (11.6, 39.7)	7196 (1.8)	32,237,054 (2.5)
Total of EMR^**^	…..	….	….	142	37.1 (34.5, 39.8)	382,367 (100)	1,283,152,555 (100)
Total of EMR^**^ without Iran	…..	….	….	101	39.7 (36.03, 42.3)	350,057 (90.6)	798,760,011 (62.2)

**a:Group1(High quality data), Group2(Medium quality of data), Group3(Low quality of data)**

^*^UAE: United Arab Emirate, ** Eastern Mediterranean Region

According to the meta-analysis of the existing reports on the ASR of BC, Lebanon had the highest rate of BC incidence, with 68.9 (95% CI, 50.4, 90.3), and Syria the lowest rate, with 24.01 (95% CI, 11.6, 39.7) per 100,000 person-year, among the 21 countries of the EMR from 2011 to 2019 (Table 2). Among EMR countries, Lebanon had the highest ASR of BC during the fourth period of the study (Supplementary File: Fig S1).

### Trend changes of breast Cancer incidence in EMR from 1998 to 2019

The pooled ASR of BC in EMR had a constant trend, followed by an increasing trend [23.04 (95% CI, 18.5, 28.1) to 23.7 (95% CI, 20.7, 26.9)] from 1998 to 2005. However, later on, it showed an upward trend to 37.1 (95% CI, 34.5, 39.8) per 100,000 person-year in the fourth period of the study (Table 3, Supplementary File: Graph S1).

**Table 3:** Comparison the ASR of Breast Cancer by Quality of Data Registration and period of the study in Eastern Mediterranean Region 1998–2019

Year	1998–2000			2001–2005			2006–2010			2011–2019		
Data	N Data point	ASR of breast cancer	I^2^ Index	N Data point	ASR of breast cancer	I^2^ Index	N Data point	ASR of breast cancer	I^2^ Index	N Data point	ASR of breast cancer	I^2^ Index
Total EMR	42	23.04 (18.5,28.1)	69.7	142	24.01 (20.2,27.04)	70.7	219	26.01 (24.01,28.1)	50.7	142	37.1(34.5, 39.8)	44.8
Total EMR without Iran	25	28.1 (20.3, 36)	78	71	36.4 (31.4, 42.3)	62.8	50	34.8 (31.4, 38.4)	19.5	101	39.7 (36.03, 42.3)	51.3
Group1: High Quality of Data	8	18.5 (12.9,24.01)	8.1	19	40.9 (31.4,51.8)	14.7	17	34.8 (28.1,42.3)	1	19	42.3 (32.5,51.8)	1.2
Group2: Medium Quality of Data	26	21.2 (16.8, 26.01)	45.7	107	18.5 (16.1, 21.2)	51.2	187	24.01 (22.1, 27.04)	50.7	74	33.6 (30.3, 37.2)	20.02
Group2: Medium Quality of Data without Iran	9	30.3 (22.1, 40.9)	44.3	36	28.1 (20.3, 36)	0	18	36.4 (31.4, 42.3)	21.1	32	34.8 (31.4, 38.4)	27.9
Group2: Iran	17	18.4 (13.7, 23.04)	34.9	71	13.7 (11.6, 16.8)	47.5	169	23.04 (18.5, 28.1)	49.3	41	31.9 (28.2, 35.8)	13.9
Group3: Low Quality of Data	8	30.3 (13.7, 53.3)	85.7	16	54.8 (37.2, 73.9)	84.9	15	33.6 (30.3, 37.2)	44.8	49	40.9 (34.8, 46.2)	63.5

### Trend changes of breast Cancer incidence in EMR according to subgroups of the quality of the data registration from 1998 to 2019

#### Countries with high quality data (group 1)

Pooled ASR of BC in the group with high data registration quality had an upward trend in the first 2 time periods [18.5 (95% CI, 12.9, 24.01) to 40.9 (95% CI, 31.4, 51.8)]. However, in the third period, the incidence of BC decreased and increased again to 42.3 (95% CI, 32.5, 51.8) per 100,000 person-year in the fourth period of the study (Table 3, Supplementary File: Graph S2).

#### Countries with medium quality of data (group 2)

Unlike the group with high data quality, a downward trend, followed by an increasing trend was observed in the group with medium data quality; the corresponding figures were 21.2 (95% CI, 16.8, 26.01) to 18.5 (95% CI, 16.1, 21.2) and to 33.6 (95% CI, 30.3, 37.21) per 100,000 person-year, respectively (Table 3, appendix 1, Graph 2). A different trend was observed after exclusion of Iran, after which the pooled ASR showed a downward trend in the first 2 period, [from 30.3 (95% CI, 22.1, 40.9) to 28.1 (95% CI, 20.3, 36)]. However, later on, it showed an upward trend to 36.4 (95% CI, 31.4, 42.3) in the third period and decreased again to 34.8(95% CI, 31.4, 38.4) per 100,000 person-year in the fourth period of the study (Table 3, Supplementary File: Graph S2).

##### Countries with low quality of data (group 3)

The trend of BC incidence in countries with low quality of data registration in EMR experienced a significant increase in the second period [30.3 (95% CI, 13.7,53.3) to 54.8 (95% CI, 37.2,73.9)]. However, in the third period, the incidence of BC decreased considerably and increased again to 40.9 (95% CI, 34.8, 46.2) per 100,000 person-year in the fourth period of the study (Table 3, Supplementary File: Graph S2).

### The association between the ASR of breast Cancer and developmental risk factors in EMR

The results of the initial analysis showed that location of the study, year of study, and quality of data had significant effects on the heterogeneity of data. The results also revealed that tau 2 [from 2.2 to 1.01] was decreased significantly after insertion of these variables in the meta-regression model (Table 4).

**Table 4:** The Association between ASR^a^ of Breast Cancer and Risk Factors

Risk Factors	Unadjusted B(95%CI)	Pvalue	Tau2	R^2^	Adjusted^b^ B(95% CI)	P value	Tau2	R^2^
HDI^c^	0.4 (−2.1, 2.9)	0.8	477.5	0	−89.2 (−119.8, −58.7)	< 0.001	352.6	26.03
TFR^d^	2.5 (0.9, 4.1)	0.002	467.2	1.8	−0.4 (−2.2, 1.4)	0.7	______	______
Obesity^e^	0.6 (0.3, 0.8)	< 0.001	455.9	4.2	1.2 (0.9, 1.5)	< 0.001	______	______
Country of study	1.5 (1.2, 1.7)	< 0.001	396.4	16.7	______	______	______	______
Year of study	1.4 (1.04, 1.8)	< 0.001	428.7	9.9	______	______	______	______
Quality of Data	−3.5 (−4.8, −2.1)	< 0.001	452.4	4.9	______	______	______	______

a:Ajusted Standard Rate of breast cancer, b: adjusted on Country of study, Year of study, Quality of Data, HDI, TFR and obesity variables, c: Human Index Development, d:Total Fertility Rate, e:Obesity = if Body Mass Index ≥ 30,

In the univariate and multivariate tests, HDI [univariate: 0.4 (95% CI, − 2.1, 2.9); multivariate: -89.2 (95% CI, − 119.8, − 58.7)] and obesity [univariate: 0.6 (95% CI, 0.3, 0.8); multivariate: 1.2 (95% CI, 0.9, 1.5)] had a significant association with BC incidence (Table 4).

## Discussion

### The current status of the ASR of breast cancer

The pooled ASR of BC occurrence was estimated to be about 37 per 100,000 person-year in EMR during 2011–2019. However, after excluding Iran, the results of sensitivity analysis showed that ASR of BC had a significant increase. The global average of BC in women was 65.5 in 2015 according to Global Burden of Disease [41] and it was 41.9 in EMR in 2012 according to GLOBOCAN [5]. The results of the present study were more consistent with GLOBOCAN 2012.

In Lebanon, the ASR of BC was almost 3 times more than that of Syria during 2011–2019. This was confirmed by another study conducted in 2004, in which the ASR of BC was higher than other Arab countries and Iran [6].

### Trend of breast Cancer incidence

There was an upward trend for the ASR of BC in EMR from 1998 to 2019. However, based on the quality of data during 1998–2019, the pattern of BC incidence has been somewhat different in EMR countries. The studies performed on Asian and EMR countries in recent years have shown an increasing trend in the occurrence of BC in these countries [48, 59, 85, 87–89, 95]. Also, WHO has predicted that the increasing trend of BC will continue in EMR countries in the next decade [84].

The highest value for the ASR of BC in the last period and the highest increase in the incidence of BC from first period to last periods of the study belonged to countries with high-quality data. In fact, only 4 countries (Kuwait, Oman, Jordan, and Tunisia) have had a population-based cancer registry prior to 1998 (11). Cancer registration systems were changed from pathological data collection to a population-based registration system during 1998–2005 in Bahrain, Qatar, Morocco, Egypt, Iran, and Libya [47]. Moreover, in addition to increase in westernized lifestyle of people living in the EMR region, cancer registry has also improved overtime [85, 96, 97]. These factors can be explained somewhat by the fluctuation in the trend of ASR of BC, with a noticeable increase in the last period. Moreover, the diversity in the increasing patterns of BC in various EMR countries may be due to the difference in the time of upgrading the cancer registry systems in these countries.

### Effects of developmental factors on breast Cancer incidence

HDI showed a negative association with the incidence of BC in the adjusted and unadjusted models, which was significant in the adjusted model; the negative association of HDI with BC incidence contradicted the findings of other studies [1, 48, 85, 98]. HDI cannot cause an increase in the incidence of BC directly; however, increased longevity, decreased fertility, increased obesity, or changes in lifestyle can increase the incidence of BC [85, 96].The highest HDI in EMR belonged to Arab countries, such as Saudi Arabia, Oman, Kuwait, Qatar, UAE, and Bahrain. The ASR of BC in these countries was lower than that of some other countries in EMR and European countries. The lower ASR of BC reported by Arab countries, compared to the Western countries, may be due to women’s higher fertility and breast-feeding. Therefore, the reverse association between HDI and ASR of BC in this study, compared to other studies, may be due to the reproductive pattern and lifestyle variety in highest HDI countries in EMR than the Western countries [99]. Lebanon, Pakistan, and Iraq, which had the highest ASR of BC during the recent years belonged to the middle, low, and middle HDI countries, respectively [11]. Several studies suggested that the higher incidence of BC in Pakistan and Iraq may be less affected by hormonal- and parity-related factors and more affected by genetic and environmental factors [100, 101]. The ASR of BC showed a positive association with obesity, as this association was confirmed by other studies [1, 48, 85, 96]. The association between ASR of BC and TFR was positive in the unadjusted and negative in the adjusted models, but it was not significant, indicating that the positive association in the unadjusted model may be due to the confounder effect of other variables, such as quality of data, location, time of the study, HDI, and obesity. Other studies have reported a significant negative association between TFR and ASR of BC [102, 103]. The difference between the findings of this study and those of others may be due to the decline in TFR in all EMR countries [17].

### Limitations of the study

Considering that no comprehensive study has been conducted on the incidence rate of BC and its trend in EMR, the results of this study can help policymakers of these countries to develop and implement programs to reduce BC incidence and improve the data registry system. However, this study had some limitations that should be taken into consideration when interpreting the results: the small number of studies; lack of availability of adequate data in some counties, especially in recent years; and the mere use of English papers. Nevertheless, in this study, it was not possible to investigate some other risk factors of BC, such as breastfeeding, diet, hormone therapy, and physical activity, which could justify the incremental trend of BC incidence. Thus, when interpreting the results of studies that have used ecological methods to investigate the association between BC incidence and risk factors, their limitations should also be taken into account.

## Conclusion

Based on the results of the study, pooled ASR of BC in EMR was lower than its global average. Also, the highest and lowest value of ASR belonged to countries with low-quality data during 2011–2018. Furthermore, there was an increasing trend in the ASR of BC in EMR in recent years, especially in the low-quality data group. The possible reasons for the incremental trend of BC incidence may be data availability and quality, or physiological factors, such as increase in the rate of obesity. Although the quality of data in cancer registry systems has improved, the published data on the incidence of BC in EMR countries have been limited in recent years. Some EMR countries still lack a national registry system or a population-based system. Thus, after development and improvements in the registry systems of EMR countries, an upward trend of BC is highly expected in this region in the future.

According to these results, it is highly recommended to investigate the factors associated with the lower incidence of breast cancer in EMR, especially in higher HDI countries in this region.

## Supplementary information

**Additional file 1: Table S1.** Strategy for systematic searches of the published literature. **Table S2.** Summarized characteristics of included studies. **Graph S1.** The ASR trend of breast cancer in Eastern Mediterian Region by qulity of data registration throughout 1998–2019. **Graph S2.** The ASR trend of breast cancer in Eastern Mediterian Region by qulity of data registration throughout 1998–2019. **Figure S1.** Geographic Distribution of ASR of breast cancer in Eastern Mediterranean Countries from 1998 to 2018.

## Data Availability

The datasets used and/or analysed during the current study are available from the corresponding author on reasonable request.

## Abbreviations

ASR: Age Standardized Rate
EMR: Eastern Mediterranean Region
Human Development Index (HDI): A metric to assess the social and economic development levels of countries. Four principal areas of examination are used to rank countries: mean years of schooling, expected years of schooling, life expectancy at birth and gross national income per capita
Obesity - adult prevalence rate: Obesity is defined as an adult having a Body Mass Index (BMI) greater to or equal to 30.0.
Total fertility rate (TFR): This entry gives a figure for the average number of children that would be born per woman if all women lived to the end of their childbearing years and bore children according to a given fertility rate at each age.
WHO: world health organization

## References

[1] Fitzmaurice C, Allen C, Barber RM, Barregard L, Bhutta ZA, Brenner H (2017). Global, regional, and national cancer incidence, mortality, years of life lost, years lived with disability, and disability-adjusted life-years for 32 cancer groups, 1990 to 2015: a systematic analysis for the global burden of disease study. JAMA Oncol.

[2] Forouzanfar MH, Foreman KJ, Delossantos AM, Lozano R, Lopez AD, Murray CJ (2011). Breast and cervical cancer in 187 countries between 1980 and 2010: a systematic analysis. Lancet..

[3] Tas F, Keskin S (2012). Age-specific incidence ratios of breast cancer (BC) in Turkey: BC in older people is increasing. Arch Gerontol Geriatr.

[4] Mehdi I, Monem EA, Al Bahrani BJ, Al Kharusi S, Nada AM, Al Lawati J (2014). Age at diagnosis of female breast cancer in Oman: issues and implications. S Asian J Cancer.

[5] Najjar H, Easson A (2010). Age at diagnosis of breast cancer in Arab nations. Int J Surg.

[6] Lakkis NA, Adib SM, Osman MH, Musharafieh UM, Hamadeh GN (2010). Breast cancer in Lebanon: incidence and comparison to regional and Western countries. Cancer Epidemiol.

[7] Karim SAM, Ghalib HHA, Mohammed SA, Fattah FHR (2015). The incidence, age at diagnosis of breast cancer in the Iraqi Kurdish population and comparison to some other countries of Middle-East and West. Int J Surg.

[8] GLOBOCAN 2018. Estimated cancer incidence, mortality and prevalence worldwide in 2018: World Health Organization; 2019.

[9] Baschieri A (2014). Health inequities in the eastern mediterranean region selected country case studies.

[10] Ghoncheh M, Soltani S, Salehiniya H (2016). Disparities in incidence and mortality of breast cancer. Iran J Public Health.

[11] Jahan S. Human development report 2016: human development for everyone: United Nations Publications; 2016.

[12] Organization WH. GLOBOCAN 2012. Estimated cancer incidence, mortality and prevalence worldwide in 2012. Breast cancer fact sheet. 2015. [Citated 2018 Aug 1] Available at http://globocaniarcfr/Pages/fact_sheets_canceraspx.

[13] Institute TJB. The Joanna Briggs Institute reviewers’ manual 2014 the systematic review of prevalence and incidence data: The Joanna Briggs Institute Reviewers’; 2014.

[14] Hoy D, Brooks P, Woolf A, Blyth F, March L, Bain C (2012). Assessing risk of bias in prevalence studies: modification of an existing tool and evidence of interrater agreement. J Clin Epidemiol.

[15] Keyfitz N (1966). Sampling variance of standardized mortality rates. Am J Hum Biol.

[16] Furukawa TA, Barbui C, Cipriani A, Brambilla P, Watanabe N (2006). Imputing missing standard deviations in meta-analyses can provide accurate results. J Clin Epidemiol.

[17] Adult prevalence rate by year chart United States of America: Central Intelligence Agency; 2018 [cited 2018. Available from: https://www.indexmundi.com/g/g.aspx?v=30&c=ir&l=en.

[18] von Bortkiewicz L (1898). Das gesetz der kleinen zahlen: BG Teubner.

[19] Iranian annual of national cancer registration report 2003-2004. Tehran: Ministry of Health Center for Disease Control& Prevention Non-Communicable Deputy Cancer Office; 2005.

[20] Iranian Annual of National Cancer Registration Report 2010-2011. Ministry of Health Center for Disease Control& Prevention Non-Communicable Deputy Cancer Office; 2013.

[21] Al-Bahrani B, Al-Busaidi A, Al-Lawati NA, Al-Siyabi NH, Al-Gharbi DO, Al-Wehaibi S (2013). Cancer incidence in Oman.

[22] Al-Madouj AN, Eldali A, Al-Zahrani AS. Ten-year cancer incidence among nationals of the GCC states 1998-2007: GCC; 2011.

[23] Al-Masri A, Kofahi S, Al-Ruhaibeh M, Al-Samen AA (2010). Epidemiology of breast cancer in the North Jordan. RMJ..

[24] Asgarian F, Mirzaei M, Asgarian S, Jazayeri M (2016). Epidemiology of breast cancer and the age distribution of patients over a period of ten years. Iran J Breast Dis.

[25] Babaei M, Mousavi S, Malek M, Tosi G, Zolfaghari M, Danaei N, et al. Cancer occurrence in Semnan Province, Iran: results of a population-based cancer registry. Asian Pac J Cancer Prev. 2005:159–64. 16101326

[26] Badar F, Mahmood S, Yusuf MA, Sultan F (2016). Epidemiology of cancers in Lahore, Pakistan, 2010–2012: a cross-sectional study. BMJ Open.

[27] Basaleem H, Al-Sakkaf K, editors. Trends of breast cancer in Aden Cancer Registry, Yemen (1997-2011): Eur J Cancer; 2016. Elsevier Sci Ltd the Boulevard, Langford Lane, Kidlington, Oxford OX5 1GB, Oxon, England.

[28] Ben Abdallah M, Ben Ayoub WH. Registre Des Cancers NORD-TUNISIE Données 2004 – 2006. Ministere De La Sante Publique Institut Salah AZAIEZ Institut National de la Santé Publique & Ministere De L’Enseignement Superieur De La Recherche Scientifique Et De La Technologie Unité de Recherche en Epidémiologie des Cancers en Tunisie.

[29] Bener A, Ayub H, Kakil R, Ibrahim W (2008). Patterns of cancer incidence among the population of Qatar: a worldwide comparative study. Asian Pac J Cancer Prev.

[30] Benider A, Harif M, Karkouri M (2012). Registre des cancers de la region du grand Casablanca (2005.2006. 2007).

[31] Bhurgri Y (2004). Karachi cancer registry data--implications for the national cancer control program of pakistan. Asian Pac J Cancer Prev.

[32] Bhurgri Y, Bhurgri H, Ajam A, Pervez S, Hasan S, Usman A (2002). Cancer patterns in Quetta (1998-1999). J Paki Med Assoc.

[33] Bhurgri Y, Naseeruddin S, Zaidi S, Bhurgri A, Zaidi Z, Hasan S (2002). Cancer patterns in Karachi division (1998-1999). J Paki Med Assoc.

[34] Bodalal Z, Azzuz R, Bendardaf R (2014). Cancers in eastern Libya: First results from Benghazi medical center. World J Gastroenterol.

[35] Bouchbika Z, Haddad H, Benchakroun N, Kotbi S, Megrini A, Bourezgui H, et al. Cancer incidence in Morocco: report from Casablanca registry 2005-2007. Pan Afr Med J. 2014;16(1). 10.11604/pamj.2013.16.31.2791 PMC3932129 24570792

[36] Curado M-P, Edwards B, Shin HR, Storm H, Ferlay J, Heanue M (2007). Cancer incidence in five continents, Volume IX: IARC Press, International Agency for Research on Cancer.

[37] Dey S, Soliman AS, Hablas A, Seifeldein IA, Ismail K, Ramadan M (2010). Urban–rural differences in breast cancer incidence in Egypt (1999–2006). Breast J.

[38] El Mistiri M, Salati M, Marcheselli L, Attia A, Habil S, Alhomri F (2015). Cancer incidence, mortality, and survival in Eastern Libya: updated report from the Benghazi Cancer Registry. Ann Epidemiol.

[39] El Mistiri M, Verdecchia A, Rashid I, El Sahli N, El Mangush M, Federico M (2007). Cancer incidence in eastern Libya: the first report from the Benghazi Cancer Registry, 2003. Int J Cancer.

[40] El-Zaemey S, Nagi N, Fritschi L, Heyworth J (2012). Breast cancer among Yemeni women using the national oncology centre registry 2004–2010. J Cancer Epidemiol.

[41] Etemad K, Goya M, Ramazani Darya Sary R, Modirian M, Parto Poor E, Arjmand Poor M (2010). Iranian annual of national cancer registration report 2007-2008.

[42] Etemad K, Goya M, Ramazani Darya Sary R, Modirian M, Parto Poor E, Arjmand Poor M (2011). Iranian annual of national cancer registration report 2008-2009.

[43] Etemad K, Goya M, Ramazani Darya Sary R, Modirian M, Parto Poor E, Arjmand Poor M (2012). Iranian annual of national cancer registration report 2009-2010.

[44] Faramarzi H, Bagheri P, Farahmandfar M, Lari MA (2013). Cancer occurrence in the south of Iran based upon pathology reports (2001–2009) Cas de cancer dans le Sud de l’Iran selon des rapports de pathologie (2001–2009). Afr J Cancer.

[45] Fararouei M, Parisai Z, Farahmand M, Haghighi RE, Toori MA (2015). Cancer incidence appears to be rising in a small province in Islamic Republic of Iran: a population-based cohort study. East Mediterr Health J.

[46] Fateh M, Emamian MH (2013). Cancer incidence and trend analysis in Shahroud, Iran, 2000-2010. Iran J Cancer Prev.

[47] Ferlay J, Bray F, Steliarova-Foucher E, Forman D. Cancer incidence in five continents, CI5plus. IARC CancerBase. 2014;(9). 10.1002/ijc.29670 26135522

[48] Fitzmaurice C, Dicker D, Pain A, Hamavid H, Moradi-Lakeh M, MacIntyre MF (2015). The global burden of cancer 2013. JAMA Oncol.

[49] Forman D, Bray F, Brewster D, Gombe Mbalawa C, Kohler B, Piñeros M (2014). Cancer incidence in five continents, vol. X (electronic version).

[50] Ghiasvand R, Adami H-O, Harirchi I, Akrami R, Zendehdel K (2014). Higher incidence of premenopausal breast cancer in less developed countries; myth or truth?. BMC Cancer.

[51] Habib OS, Al-Diab J, Mohsin AA, Al-Elwe W, Hasan JG, Al-Haroon SS (2010). Experience and outcome of population-based cancer registration in Basrah-Southern Iraq in 2005-2008. Asian Pac J Cancer Prev.

[52] Habib OS, Hameed LA, Ajeel NA, Al-Hawaz MH, Al-Faddagh ZA, Nasr GN (2016). Epidemiology of breast cancer among females in Basrah. Asian Pac J Cancer Prev.

[53] Hagh Azali M, Ramazani Darya Sary R, DonLoo M, Sedighy Z, Dabiri E, Parto Poor E. Iranian annual of national cancer registration report 2002007-6. Tehran: Ministry of Health Center for Disease Control& Prevention Non-Communicable Deputy Cancer Office; 2008.

[54] Hanif M, Sabeen B, Maqbool A, Ahmed A, Nadeem F, Habib S (2015). Breast cancer: incidence (Thirteen year data analysis) and one year clinicopathological data of patients in a tertiary care cancer hospital. Int J Biol Biotechnol.

[55] Hanif M, Zaidi P, Kamal S, Hameed A (2009). Institution-based cancer incidence in a local population in Pakistan: nine year data analysis. Asian Pac J Cancer Prev.

[56] Hashemzadeh S, Maleki RA, Golzari SE (2012). The incidence of breast cancer in northwest iran (2003-2008). J Cardiovasc Thorac Res.

[57] Hussain A, Ahmad SB, Muhammad W, Kakakhail M, Matiullah (2008). Epidemiology of the breast cancer patients registered at Institute of Radiotherapy and Nuclear Medicine, Peshawar, Pakistan. Eur J Cancer Care.

[58] Ibrahim AS, Khaled HM, Mikhail NN, Baraka H, Kamel H. Cancer incidence in Egypt: results of the national population-based cancer registry program. J Cancer Epidemiol. 2014;2014. 10.1155/2014/437971 PMC4189936 25328522

[59] Ismail SI, Soubani M, Nimri JM, Al-Zeer AH (2013). Cancer incidence in Jordan from 1996 to 2009: a comprehensive study. Asian Pac J Cancer Prev.

[60] Khoshnaw N, Mohammed HA, Abdullah DA (2015). Patterns of cancer in Kurdistan-results of eight years cancer registration in Sulaymaniyah Province-Kurdistan-Iraq. Asian Pac J Cancer Prev.

[61] Majid RA, Mohammed HA, Hassan HA, Abdulmahdi WA, Rashid RM, Hughson MD (2012). A population-based study of Kurdish breast cancer in northern Iraq: hormone receptor and HER2 status. A comparison with Arabic women and United States SEER data. BMC Womens Health.

[62] Mashhadi M, Zakeri Z, Abdollahinejad M (2010). Cancer incidence in South East of Iran: results of a population-based cancer registry. Shiraz E Med J.

[63] Masoompour SM, Lankarani KB, Honarvar B, Tabatabaee SH, Moghadami M, Khosravizadegan Z (2016). Changing epidemiology of common cancers in southern iran, 2007-2010: A cross sectional study. PLoS One.

[64] Mehrabani D, Almasi A, Farahmand M, Ahrari Z, Rezaianzadeh A, Mehrabani G (2012). Incidence of breast cancer in Fars province, southern Iran: a hospital-based study. World J Plast Surg.

[65] Mohagheghi S, Mousavi JS, Malekzadeh R, Parkin M (2009). Cancer incidence in Tehran metropolis: the first report from the Tehran population-based cancer registry, 1998–2001.

[66] Moosavi S, DonLoo M, Hajsadeghi N, Seddighi Z, Rahmani Basir S, Jalal R, et al. Iranian annual of national cancer registration report 2005-2006: Ministry of Health Center for Disease Control& Prevention Non-Communicable Deputy Cancer Office; 2007.

[67] Moradpour F, Fatemi Z (2013). Estimation of the projections of the incidence rates, mortality and prevalence due to common cancer site in Isfahan, Iran. Asian Pac J Cancer Prev.

[68] Mousavi SM, Mohaghegghi MA, Mousavi-Jerrahi A, Nahvijou A, Seddighi Z (2006). Burden of breast cancer in Iran: a study of the Tehran population based cancer registry. Asian Pac J Cancer Prev.

[69] Mzayek F, Asfar T, Rastam S, Maziak W (2002). Neoplastic diseases in Aleppo, Syria. Eur J Cancer Prev.

[70] Nimri O, Al-Sayaideh A (2012). Cancer incidence in Jordan.

[71] Qureshi MA, Mirza T, Khan S, Sikandar B, Zahid M, Aftab M (2016). Cancer patterns in Karachi (all districts), Pakistan: first results (2010–2015) from a pathology based cancer registry of the largest government-run diagnostic and reference center of Karachi. Cancer Epidemiol.

[72] Ramazani Darya Sary R, Moosavi S, DonLoo M, Hajsadeghi N, Khoshideh MM, Moradpour F (2006). Iranian annual of national cancer registration report 2004-2005.

[73] Sadjadi A, Nouraie M, Ghorbani A, Alimohammadian M, Malekzadeh R (2009). Epidemiology of breast cancer in the Islamic Republic of Iran: first results from a population-based cancer registry.

[74] Sadjadi A, Zahedi M, Nouraie M, Alimohammadian M, Ghorbani A, Bahmanyar S (2007). The first population-based cancer survey in Kerman Province of Iran. Iran J Public Health.

[75] Saeed IE, Weng HY, Mohamed KH, Mohammed SI (2014). Cancer incidence in Khartoum, Sudan: first results from the cancer registry, 2009–2010. Cancer Med.

[76] Sellami A, Sellami Boudawara T. Incidence des cancers dans le Gouvernorat de Sfax: 2000–2002: Institut National de la Santé Publique; 2007.

[77] Shamseddine A, Sibai A-M, Gehchan N, Rahal B, El-Saghir N, Ghosn M (2004). Cancer incidence in postwar Lebanon: findings from the first national population-based registry, 1998∗. Ann Epidemiol.

[78] Somi MH, Farhang S, Mirinezhad SK, Naghashi S, Seif-Farshad M, Golzari M (2008). Cancer in East Azerbaijan, Iran: results of a population-based cancer registry. Asian Pac J Cancer Prev.

[79] Taheri NS, Nosrat SB, Aarabi M, Tabiei MN, Kashani E, Rajaei S (2012). Epidemiological pattern of breast cancer in Iranian women: is there an ethnic disparity?. Asian Pac J Cancer Prev.

[80] Tazi MA, Benjaafar N, Er-Raki A. Incidence des cancers a Rabat – anne’e2005: Assocition scientifique de l^,^Institu National d^,^oncologie (ASINO) & Direction deL^,^Epidemiologie et de lutte contre les Maladies (DELM); 2009.

[81] Tazi MA, Er-Raki A, Benjaafar N. Cancer incidence in Rabat, Morocco: 2006–2008. Ecancermedicalscience. 2013;7. 10.3332/ecancer.2013.338 PMC3737118 23940493

[82] Yavari P, Hislop TG, Bajdik C, Sadjadi A, Nouraie M, Babai M (2006). Comparison of cancer incidence in Iran and Iranian immigrants to British Columbia, Canada. Asian Pac J Cancer Prev.

[83] Zanetti R, Tazi MA, Rosso S (2010). New data tells us more about cancer incidence in North Africa. Eur J Cancer.

[84] Organization WH (2009). Towards a strategy for cancer control in the Eastern Mediterranean Region.

[85] Ghoncheh M, Mohammadian-Hafshejani A, Salehiniya H (2015). Incidence and mortality of breast cancer and their relationship to development in Asia. Asian Pac J Cancer Prev.

[86] Hamadeh RR, Abulfatih NM, Fekri MA, Al-Mehza HE (2014). Epidemiology of breast cancer among Bahraini women: data from the Bahrain cancer registry. Sultan Qaboos Univ Med J.

[87] Al-Hashimi M, Wang XJ (2014). Breast cancer in Iraq, incidence trends from 2000-2009. Asian Pac J Cancer Prev.

[88] Jazayeri SB, Saadat S, Ramezani R, Kaviani A (2015). Incidence of primary breast cancer in Iran: Ten-year national cancer registry data report. Cancer Epidemiol.

[89] Missaoui N, Trabelsi A, Parkin DM, Jaidene L, Chatti D, Mokni M (2010). Trends in the incidence of cancer in the Sousse region, Tunisia, 1993–2006. Int J Oncol.

[90] Shamseddine A, Saleh A, Charafeddine M, Seoud M, Mukherji D, Temraz S (2014). Cancer trends in Lebanon: a review of incidence rates for the period of 2003–2008 and projections until 2018. Popul Health Metrics.

[91] Roshandel, Reza G, Motlagh G, Ali PP, Elham S, Fereshteh Y, Majid (2018). Annual report of Iranian national population-based cancer registry (2014).

[92] Fazel A, Hasanpour-Heidari S, Salamat F, Rajaie S, Kazeminezhad V, Naeimi-Tabiei M (2019). Marked increase in breast cancer incidence in young women: A 10-year study from Northern Iran, 2004-2013. Cancer Epidemiol.

[93] Roshandel G, Ghanbari-Motlagh A, Partovipour E, Salavati F, Hasanpour-Heidari S, Mohammadi G (2019). Cancer incidence in Iran in 2014: Results of the Iranian National Population-based Cancer Registry. Cancer Epidemiol.

[94] Sharma R (2019). Breast cancer incidence, mortality and mortality-to-incidence ratio (MIR) are associated with human development, 1990-2016: evidence from Global Burden of Disease Study 2016. Breast Cancer (Tokyo, Japan).

[95] Zahmatkesh B, Keramat A, Alavi N, Khosravi A, Kousha A, Motlagh AG (2016). Breast cancer trend in Iran from 2000 to 2009 and prediction till 2020 using a trend analysis method. Asian Pac J Cancer Prev.

[96] DeSantis CE, Bray F, Ferlay J, Lortet-Tieulent J, Anderson BO, Jemal A. International variation in female breast cancer incidence and mortality rates. Cancer Epidemiol Biomark Prev. 2015. 10.1158/1055-9965.EPI-15-0535 26359465

[97] Youlden DR, Cramb SM, Dunn NA, Muller JM, Pyke CM, Baade PD (2012). The descriptive epidemiology of female breast cancer: an international comparison of screening, incidence, survival and mortality. Cancer Epidemiol.

[98] Moraga-Serrano PE. Global, regional, and national cancer incidence, mortality, years of life lost, years lived with disability, and disability-adjusted life-years for 29 cancer groups, 1990 to 2016: a systematic analysis for the global burden of disease study. JAMA Oncol. 2018. 10.1001/jamaoncol.2018.2706 PMC6248091 29860482

[99] Corbex M, Harford JB (2013). Perspectives on breast cancer in Arab populations. Lancet Oncol.

[100] Zahra F, Humayoun F, Yousaf T, Khan NA. Evaluation of risk factors for carcinoma breast in Pakistani women. J Fatima Jinnah Med Univ. 2013;7(1).

[101] Asif HM, Sultana S, Akhtar N, Rehman JU, Rehman RU (2014). Prevalence, risk factors and disease knowledge of breast cancer in Pakistan. Asian Pac J Cancer Prev.

[102] Rosero‐Bixby L, Oberle MW, Lee NC (1987). Reproductive history and breast cancer in a population of high fertility, Costa Rica, 1984–85. Int J Cancer.

[103] Madigan MP, Ziegler RG, Benichou J, Byrne C, Hoover RN (1995). Proportion of breast cancer cases in the United States explained by well-established risk factors. JNCI.

