# Supplementary material for: Incidence trend of breast Cancer in women of eastern Mediterranean region countries from 1998 to 2019: A systematic review and meta-analysis
Source: BMC Womens Health. 2020 Mar 17;20:53. doi: 10.1186/s12905-020-00903-z (PMC7079343; doi:10.1186/s12905-020-00903-z)
Supplement: Supplementary file 1 — Additional file 1: Table S1. Strategy for systematic searches of the published literature. Table S2. Summarized characteristics of included studies. Graph S1. The ASR trend of breast cancer in Eastern Mediterian Region by qulity of data registration throughout 1998–2019. Graph S2. The ASR trend of breast cancer in Eastern Mediterian Region by qulity of data registration throughout 1998–2019. Figure S1. Geographic Distribution of ASR of breast cancer in Eastern Mediterranean Countries from 1998 to 2018. [file 12905_2020_903_MOESM1_ESM.docx]

**Supplementary Files**

**Table S1: Strategy for systematic searches of the published literature**

**Search ((((((((("breast cancer") OR "breast tumor") OR "breast neoplasms") OR (("breast") AND "tumor"))) OR (("breast") AND "cancer" ))) OR (("breast") AND "neoplasms" AND ((((((("frequency") OR "incidence") OR "prevalence") OR "epidemiology") OR "distribution") OR "supply"))) AND (((((((((((((((((((((("Eastern Mediterranean Region") OR Somalia) OR "United Arab Emirates") OR Djibouti) OR Tunisia) OR Syria) OR Sudan) OR Saudi Arabia) OR Morocco) OR Libya) OR Bahrain) OR Iraq) OR Yemen) OR Jordan) OR Oman) OR Lebanon) OR Egypt) OR Qatar) OR afghanistan) OR kuwait) OR pakistan) OR "iran")**

**Table S2: Summarized characteristics of included studies**

| **Author** | **Year of publish Data** | **Country** | **City** | **Duration of study** | **Source of Data** | **Year of Data** | **ASR** | **SE ASR** | **ICR** | **SE ICR** | **Total Breast cases** |
| --- | --- | --- | --- | --- | --- | --- | --- | --- | --- | --- | --- |
| A. Al-Masri,[22] | 2010 | Jordan | North Jordan | 2007 | Hospital | 2007 | 24.3 | _____ | _____ | _____ | 36 |
| A. Bener, [27] | 2008 | Qatar | Qatar | 1991-2006 | Hospital | 2006 | 30.1 | _____ | _____ | _____ | 770 |
| A. Benider,[28] | 2012 | Morocco | Casablanca | 2005-2007 | Cancer Registry | 2005 | 35.1 | _____ | _____ | _____ | 666 |
| A. Benider, [28] | 2012 | Morocco | Casablanca | 2005-2007 | Cancer Registry | 2006 | 35.4 | _____ | _____ | _____ | 690 |
| A. Benider, [28] | 2012 | Morocco | Casablanca | 2005-2007 | Cancer Registry | 2007 | 38.6 | _____ | _____ | _____ | 763 |
| A. Hussain, [51] | 2008 | Pakistan | Peshawar | 1995-2001 | Radiotherapy | 1998 | 2.04 | _____ | 2.04 | _____ | 2059 |
| A. S. Ibrahim, [52] | 2014 | Egypt | Egypt | 2008-2011 | Cancer Registry | 2010 | 48.8 | _____ | 35.8 | _____ | _____ |
| A. Sadiadi, [67] | 2007 | Iran | Iran | 2000-2002 | Globo can | 2000 | 35.7 | 1.42 | _____ | _____ | _____ |
| A. Sadiadi, [67] | 2007 | Iran | Iran | 2002 | Globo can | 2002 | 17.1 | 1.084 | _____ | _____ | _____ |
| A. Sadiadi, [66] | 2009 | Iran | Kerman | 1996-2000 | Cancer Registry | 1998 | 16.9 | 0.71 | 11.2 | 0.04 | 554 |
| A. Sadiadi,[66] | 2009 | Iran | Gilan,Mazandaran,Kerman,Golestan,Ardabil | 1996-2000 | Cancer Registry | 1998 | 16.2 | 0.42 | 4.7 | 0.09 | 2421 |
| A. Sadiadi, [66] | 2009 | Iran | Ardabil | 1996-2000 | Cancer Registry | 1998 | 7.6 | 0.73 | 10.1 | 0.04 | 106 |
| A. Sadiadi,[66] | 2009 | Iran | Mazandran | 1996-2000 | Cancer Registry | 1998 | 23.9 | 0.79 | 11.6 | 0.05 | 899 |
| A. Sadiadi,[66] | 2009 | Iran | Gilan | 1996-2000 | Cancer Registry | 1998 | 12.6 | 0.57 |  |  | 513 |
| A. Sadiadi,[66] | 2009 | Iran | Golestan | 1996-2000 | Cancer Registry | 1998 | 15.7 | 0.82 | 20.5 | 0.03 | 349 |
| A. SELLAMI, [69] | 2007 | Tunisia | Safix | 2000-2002 | Cancer Registry | 2001 | 28 | 1.60 | 25.2 |  | 305 |
| A. Shamseddine,[80] | 2014 | Lebanon | Lebanon | 2003-2008 | Cancer Registry | 2003 | 78.3 | 2.2 | 69 | 0.02 | 1242 |
| A. Shamseddine,[80] | 2014 | Lebanon | Lebanon | 2003-2008 | Cancer Registry | 2008 | 95.7 | 2.202 |  |  |  |
| A. Shamseddine[70] | 2004 | Lebanon | Lebanon | 1988 | Cancer Registry | 1998 | 46.7 | 1.76 | 42.3 | 0.03 | 698 |
| C. Fitzmaurice,[1] | 2017 | Iran | Iran | 2005-2015 | Cancer Registry & Model Estimation | 2005 | 30.6 | 3.6 | _____ | _____ | 7112 |
| C. Fitzmaurice,[1] | 2017 | Iran | Iran | 2005-2015 | Cancer Registry & Model Estimation | 2015 | 36 | 5.5 | _____ | _____ | 11804 |
| C. Fitzmaurice,[93] | 2015 | Iran | Iran | 1990-2013 | Cancer Registry & Model Estimation | 2013 | 17.9 | 2.54 | 5.9 | 0.02 | 2590 |
| O. Nimri, [89] |  | Jordan | Jordan | 2000-2012 | Cancer Registry | 2000 | 41 | _____ | _____ | _____ | 551 |
| O. Nimri, [89] |  | Jordan | Jordan | 2000-2012 | Cancer Registry | 2001 | 35.5 | _____ | _____ | _____ | 566 |
| O. Nimri, [89] |  | Jordan | Jordan | 2000-2012 | Cancer Registry | 2002 | 30.8 | _____ | _____ | _____ | 501 |
| O. Nimri, [89] |  | Jordan | Jordan | 2000-2012 | Cancer Registry | 2003 | 32.2 | _____ | _____ | _____ | 551 |
| O. Nimri, [89] |  | Jordan | Jordan | 2000-2012 | Cancer Registry | 2004 | 40.1 | _____ | _____ | _____ | 646 |
| O. Nimri, [89] |  | Jordan | Jordan | 2000-2012 | Cancer Registry | 2005 | 41.4 | _____ | _____ | _____ | 674 |
| O. Nimri, [89] |  | Jordan | Jordan | 2000-2012 | Cancer Registry | 2006 | 45.6 | _____ | _____ | _____ | 749 |

| **Author** | **Year of publish Data** | **Country** | **City** | **Duration of study** | **Source of Data** | **Year of Data** | **ASR** | **SE ASR** | **ICR** | **SE ICR** | **Total Breast cases** |
| --- | --- | --- | --- | --- | --- | --- | --- | --- | --- | --- | --- |
| O. Nimri, [89] |  | Jordan | Jordan | 2000-2012 | Cancer Registry | 2007 | 48.9 | _____ | _____ | _____ | 817 |
| O. Nimri, [89] |  | Jordan | Jordan | 2000-2012 | Cancer Registry | 2008 | 50.2 | _____ | _____ | _____ | 855 |
| O. Nimri, [89] |  | Jordan | Jordan | 2000-2012 | Cancer Registry | 2009 | 53.8 | _____ | _____ | _____ | 926 |
| O. Nimri, [89] |  | Jordan | Jordan | 2000-2012 | Cancer Registry | 2010 | 52.7 | _____ | _____ | _____ | 941 |
| O. Nimri, [89] |  | Jordan | Jordan | 2000-2012 | Cancer Registry | 2011 | 51.3 | _____ | _____ | _____ | 935 |
| O. Nimri, [89] |  | Jordan | Jordan | 2000-2012 | Cancer Registry | 2012 | 53.9 | _____ | _____ | _____ | 994 |
| D. B. Al-Bahrani;,[20] |  | Oman | Oman | 1998-2000 | Cancer Registry | 1999 | 14.6 | _____ | _____ | _____ | 265 |
| D. B. Al-Bahrani;,[20] |  | Oman | Oman | 2001-2013 | Cancer Registry | 2001 | 16 | _____ | _____ | _____ | 77 |
| D. B. Al-Bahrani;,[20] |  | Oman | Oman | 2001-2013 | Cancer Registry | 2002 | 14.8 | _____ | _____ | _____ | 74 |
| D. B. Al-Bahrani;,[20] |  | Oman | Oman | 2001-2013 | Cancer Registry | 2003 | 13.6 | _____ | _____ | _____ | 63 |
| D. B. Al-Bahrani;,[20] |  | Oman | Oman | 2001-2013 | Cancer Registry | 2004 | 22.9 | _____ | _____ | _____ | 95 |
| D. B. Al-Bahrani;,[20] |  | Oman | Oman | 2001-2013 | Cancer Registry | 2005 | 21.8 | _____ | _____ | _____ | 95 |
| D. B. Al-Bahrani;,[20] |  | Oman | Oman | 2001-2013 | Cancer Registry | 2006 | 22.6 | _____ | _____ | _____ | 97 |
| D. B. Al-Bahrani;,[20] |  | Oman | Oman | 2001-2013 | Cancer Registry | 2007 | 22.1 | _____ | _____ | _____ | 105 |
| D. B. Al-Bahrani;,[20] |  | Oman | Oman | 2001-2013 | Cancer Registry | 2008 | 22 | _____ | _____ | _____ | 106 |
| D. B. Al-Bahrani;,[20] |  | Oman | Oman | 2001-2013 | Cancer Registry | 2009 | 22.2 | _____ | _____ | _____ | 118 |
| D. B. Al-Bahrani;,[20] |  | Oman | Oman | 2001-2013 | Cancer Registry | 2010 | 24.2 | _____ | _____ | _____ | 134 |
| D. B. Al-Bahrani;,[20] |  | Oman | Oman | 2001-2013 | Cancer Registry | 2011 | 27.7 | _____ | _____ | _____ | 160 |
| D. B. Al-Bahrani;,[20] |  | Oman | Oman | 2001-2013 | Cancer Registry | 2012 | 22.8 | _____ | _____ | _____ | 149 |
| D. B. Al-Bahrani;,[20] |  | Oman | Oman | 2001-2013 | Cancer Registry | 2013 | 22 | _____ | _____ | _____ | 154 |
| D. Forman, [44] | 2014 | Egypt | Gharbiah | 2003-2007 | IARC | 2005 | 45.4 | _____ | 37.8 | _____ | 3675 |
| D. Forman, [44] | 2014 | Kuwait | Kuwait | 2003-2007 | IARC | 2005 | 46 | _____ | 28.2 | _____ | 1179 |
| D. Forman, [44] | 2014 | Libya | Benghazi | 2003-2005 | IARC | 2004 | 22.9 | _____ | 16 | _____ | 364 |
| D. Forman, [44] | 2014 | Saudi Arabia | Saudi Arabia | 2003-2007 | IARC | 2005 | 21.1 | _____ | 12.6 | _____ | 1179 |
| D. Forman, [44] | 2014 | Tunisia North | Tunisia North | 2003-2006 | IARC | 2004 | 30.9 | _____ | 31.3 | _____ | 2260 |
| D. Mehrabani, [58] | 2012 | Iran | Fars | 2001-2006 | Cancer Registry | 2003 | 19.1 | _____ | 15.4 | 0.022428 | 1988 |
| F. Asgarian, [23] | 2016 | Iran | Kashan | 2001-2011 | Cancer Registry | 2001 | 20.2 | _____ | _____ | _____ | 34 |
| F. Asgarian, [23] | 2016 | Iran | Kashan | 2001-2011 | Cancer Registry | 2011 | 32.8 | _____ | _____ | _____ | 52 |
| F. Badar, [94] | 2016 | Pakistan | Lahor | 2010-2012 | Hospital | 2011 | 476 | _____ | 29.2 | _____ | 4082 |

| **Author** | **Year of publish** **DATA** | **Country** | **City** | **Duration of study** | **Source of Data** | **Year of Data** | **ASR** | **SE ASR** | **ICR** | **SE ICR** | **Total Breast cases** |
| --- | --- | --- | --- | --- | --- | --- | --- | --- | --- | --- | --- |
| F. Moradpour [61] | 2013 | Iran | Isfahan | 2001-2010 | Cancer Registry | 2001 | 10.7 | _____ | 9.2 | _____ | 190 |
| F. Moradpour [61] | 2013 | Iran | Isfahan | 2001-2010 | Cancer Registry | 2010 | 46.7 | _____ | 41.4 | _____ | 944 |
| F. Mzayek, [63] | 2002 | Syria | Syria | 1998-1999 | Pathology | 1999 | 30.4 | 2 | _____ | _____ | 230 |
| H. Basaleem [25] | 2016 | Yemen | Aden | 1997-2011 | Cancer Registry | 2004 | 11 | 0.33 | _____ | 0.03 | 1107 |
| H. Faramarzi, [40] | 2013 | Iran | Fars | 2001-2009 | Cancer Registry | 2005 | 112.9 | 2.29 | 99.7 | 0.02 | 2430 |
| I. E. Saeed, [68] | 2014 | Sudan | Khartoum | 2009-2010 | Cancer Registry | 2010 | 41.1 | 1.23 | 3 | 0.03 | 1112 |
| N. Missaoui,[79] | 2010 | Tunisia | Sousse | 1998-2002 | Cancer Registry | 2000 | 29.8 | _____ | 25.2 | _____ | 308 |
| N. Missaoui,[79] | 2010 | Tunisia | Sousse | 2003-2006 | Cancer Registry | 2005 | 28.3 | _____ | 25.5 | _____ | 277 |
| N. S. Taheri, [72] | 2012 | Iran | Golestan | 2004-2009 | Cancer Registry | 2006 | 28 | _____ | _____ | _____ | 1101 |
| O. S. Habib, [46] | 2010 | Iraq | Basra | 2005-2008 | Cancer Registry | 2007 | 32.6 | _____ | _____ | _____ | 1030 |
| P. Yavari, [75] | 2006 | Iran | Ardabil | 1996-2000 | Cancer Registry | 1998 | 7.6 | 1.8 | _____ | _____ | _____ |
| P. Yavari,[75] | 2006 | Iran | Kerman | 19962000 | Cancer Registry | 1998 | 16.9 | 2.4 | _____ | _____ | _____ |
| R. A. Majid, [55] | 2012 | Iraq | Iraq | 2008-2010 | Hospital | 2009 | 40.5 | _____ | 1.64 | _____ | 824 |
| R. Ghiasvand,[45] | 2014 | Pakistan | Pakistan | 2008 | Globo can | 2008 | 31.5 | _____ | 21.77 | _____ | 19271 |
| R. Ghiasvand,[45] | 2014 | Egypt | Egypt | 2008 | Globo can | 2008 | 37.3 | _____ | 30.19 | _____ | 12621 |
| R. R. Hamadeh, [76] | 2014 | Bahrain | Bahrain | 2000-2010 | Cancer Registry | 2000 | 58.2 | _____ | 17.95 | _____ | 91 |
| R. R. Hamadeh, [76] | 2014 | Bahrain | Bahrain | 2000-2010 | Cancer Registry | 2001 | 64.9 | _____ | 17.95 | _____ | 91 |
| R. R. Hamadeh, [76] | 2014 | Bahrain | Bahrain | 2000-2010 | Cancer Registry | 2002 | 42.6 | _____ | 17.95 | _____ | 66 |
| R. R. Hamadeh, [76] | 2014 | Bahrain | Bahrain | 2000-2010 | Cancer Registry | 2003 | 51.8 | _____ | 17.95 | _____ | 91 |
| R. R. Hamadeh, [76] | 2014 | Bahrain | Bahrain | 2000-2010 | Cancer Registry | 2004 | 54.8 | _____ | 17.95 | _____ | 91 |
| R. R. Hamadeh, [76] | 2014 | Bahrain | Bahrain | 2000-2010 | Cancer Registry | 2005 | 59.9 | _____ | 17.95 | _____ | 91 |
| R. R. Hamadeh, [76] | 2014 | Bahrain | Bahrain | 2000-2010 | Cancer Registry | 2006 | 57.5 | _____ | 17.95 | _____ | 91 |
| R. R. Hamadeh, [76] | 2014 | Bahrain | Bahrain | 2000-2010 | Cancer Registry | 2007 | 58.2 | _____ | 17.95 | _____ | 120 |
| R. R. Hamadeh, [76] | 2014 | Bahrain | Bahrain | 2000-2010 | Cancer Registry | 2008 | 45.2 | _____ | 17.95 | _____ | 91 |
| R. R. Hamadeh, [76] | 2014 | Bahrain | Bahrain | 2000-2010 | Cancer Registry | 2009 | 38 | _____ | 17.95 | _____ | 91 |
| R. R. Hamadeh, [76] | 2014 | Bahrain | Bahrain | 2000-2010 | Cancer Registry | 2010 | 44.4 | _____ | 17.95 | _____ | 91 |
| R. Zanetti, [90] | 2010 | Morocco | Casablanca | 2004 | Cancer Registry | 2004 | 35 | _____ | _____ | _____ | _____ |
| R. Zanetti, [90] | 2010 | Morocco | Rabat | 2005 | Cancer Registry | 2005 | 35.8 | _____ | _____ | _____ | _____ |
| R. Zanetti, [90] | 2010 | Tunisia | Northern | 1999-2003 | Cancer Registry | 2001 | 29.6 | _____ | _____ | _____ | _____ |
| R. Zanetti, [90] | 2010 | Egypt | Gharbiah | 1999-2002 | Cancer Registry | 2000 | 42.5 | _____ | _____ | _____ | _____ |
| S. B. Jazayeri, [78] | 2015 | Iran | Iran | 2000-2010 | Cancer Registry | 2005 | 22.86 | _____ | 22.4 | _____ | 50590 |
| S. B. Jazayeri, [78] | 2015 | Iran | Tabriz | 2000-2010 | Cancer Registry | 2005 | 21.8 | _____ | 22.7 | _____ | _____ |
| S. B. Jazayeri, [78] |  |  |  |  |  |  |  |  |  |  | _____ |

| **Author** | **Year of publish** **Data** | **Country** | **City** | **Duration of study** | **Source of Data** | **Year of Data** | **ASR** | **SE ASR** | **ICR** | **SE ICR** | **Total Breast cases** |
| --- | --- | --- | --- | --- | --- | --- | --- | --- | --- | --- | --- |
| [18] | 2005 | Iran | Ardabil | 2003-2004 | Cancer Registry | 2004 | 6.1 | _____ | _____ | _____ | _____ |
| [18] | 2005 | Iran | Azarbaijan gharbi | 2003-2004 | Cancer Registry | 2004 | 8.3 | _____ | _____ | _____ | _____ |
| [18] | 2005 | Iran | Azarbaijan sharghi | 2003-2004 | Cancer Registry | 2004 | 4.9 | _____ | _____ | _____ | _____ |
| [18] | 2005 | Iran | Booshehr | 2003-2004 | Cancer Registry | 2004 | 11.4 | _____ | _____ | _____ | _____ |
| [18] | 2005 | Iran | Chaharmahal bakhtiari | 2003-2004 | Cancer Registry | 2004 | 2.89 | _____ | _____ | _____ | _____ |
| [18] | 2005 | Iran | Isfahan | 2003-2004 | Cancer Registry | 2004 | 17.3 | _____ | _____ | _____ | _____ |
| [18] | 2005 | Iran | Fars | 2003-2004 | Cancer Registry | 2004 | 16.5 | _____ | _____ | _____ | _____ |
| [18] | 2005 | Iran | Ghazvin | 2003-2004 | Cancer Registry | 2004 | 9.6 | _____ | _____ | _____ | _____ |
| [18] | 2005 | Iran | Gilan | 2003-2004 | Cancer Registry | 2004 | 12.9 | _____ | _____ | _____ | _____ |
| [18] | 2005 | Iran | Golestan | 2003-2004 | Cancer Registry | 2004 | 8.4 | _____ | _____ | _____ | _____ |
| [18] | 2005 | Iran | Hamadan | 2003-2004 | Cancer Registry | 2004 | 7.8 | _____ | _____ | _____ | _____ |
| [18] | 2005 | Iran | Hormozgan | 2003-2004 | Cancer Registry | 2004 | 4.3 | _____ | _____ | _____ | _____ |
| [18] | 2005 | Iran | Ilam | 2003-2004 | Cancer Registry | 2004 | 3.7 | _____ | _____ | _____ | _____ |
| [18] | 2005 | Iran | Kerman | 2003-2004 | Cancer Registry | 2004 | 11.3 | _____ | _____ | _____ | _____ |
| [18] | 2005 | Iran | Kermanshah | 2003-2004 | Cancer Registry | 2004 | 25.1 | _____ | _____ | _____ | _____ |
| [18] | 2005 | Iran | Khorasan rezavi | 2003-2004 | Cancer Registry | 2004 | 17.6 | _____ | _____ | _____ | _____ |
| [18] | 2005 | Iran | Khozestan | 2003-2004 | Cancer Registry | 2004 | 15.02 | _____ | _____ | _____ | _____ |
| [18] | 2005 | Iran | Kordestan | 2003-2004 | Cancer Registry | 2004 | 3.5 | _____ | _____ | _____ | _____ |
| [18] | 2005 | Iran | Lorestan | 2003-2004 | Cancer Registry | 2004 | 5.8 | _____ | _____ | _____ | _____ |
| [18] | 2005 | Iran | Markazi | 2003-2004 | Cancer Registry | 2004 | 7.5 | _____ | _____ | _____ | _____ |
| [18] | 2005 | Iran | Mazandaran | 2003-2004 | Cancer Registry | 2004 | 12.3 | _____ | _____ | _____ | _____ |
| [18] | 2005 | Iran | Qom | 2003-2004 | Cancer Registry | 2004 | 16.1 | _____ | _____ | _____ | _____ |
| [18] | 2005 | Iran | semnan | 2003-2004 | Cancer Registry | 2004 | 10.1 | _____ | _____ | _____ | _____ |
| [18] | 2005 | Iran | Sistan and Baluchestan | 2003-2004 | Cancer Registry | 2004 | 3.1 | _____ | _____ | _____ | _____ |
| [18] | 2005 | Iran | Tehran | 2003-2004 | Cancer Registry | 2004 | 36.9 | _____ | _____ | _____ | _____ |
| [18] | 2005 | Iran | Yazd | 2003-2004 | Cancer Registry | 2004 | 31.22 | _____ | _____ | _____ | _____ |
| [18] | 2005 | Iran | zanjan | 2003-2004 | Cancer Registry | 2004 | 5.4 | _____ | _____ | _____ | _____ |
| R. Ramazani Darya Sary, [65] | 2006 | Iran | Ardabil | 2004-2005 | Cancer Registry | 2005 | 7.8 | _____ | 6.876082 | _____ | 42 |
| R. Ramazani Darya Sary, [65] | 2006 | Iran | Azarbaijan gharbi | 2004-2005 | Cancer Registry | 2005 | 13.1 | _____ | 9.684183 | _____ | 137 |
| R. Ramazani Darya Sary, [65] | 2006 | Iran | Azarbaijan sharghi | 2004-2005 | Cancer Registry | 2005 | 7.6 | _____ | 10.20342 | _____ | 180 |

| **Author** | | **Year of publish** **Data** | | **Country** | | **City** | | **Duration of study** | | **Source of Data** | | **Year of Data** | | **ASR** | | **SE ASR** | | **ICR** | | **SE ICR** | | **Total Breast cases** | |
| --- | --- | --- | --- | --- | --- | --- | --- | --- | --- | --- | --- | --- | --- | --- | --- | --- | --- | --- | --- | --- | --- | --- | --- |
| R. Ramazani Darya Sary, [65] | | 2006 | | Iran | | Booshehr | | 2004-2005 | | Cancer Registry | | 2005 | | 8.5 | | _____ | | 10.76 | | _____ | | 45 | |
| R. Ramazani Darya Sary, [65] | | 2006 | | Iran | | Chaharmahal bakhtiari | | 2004-2005 | | Cancer Registry | | 2005 | | 6.1 | | _____ | | 6.8 | | _____ | | 29 | |
| R. Ramazani Darya Sary, [65] | | 2006 | | Iran | | Isfahan | | 2004-2005 | | Cancer Registry | | 2005 | | 20.9 | | _____ | | 15.42 | | _____ | | 343 | |
| R. Ramazani Darya Sary, [65] | | 2006 | | Iran | | Fars | | 2004-2005 | | Cancer Registry | | 2005 | | 19.04 | | _____ | | 11.4 | | _____ | | 243 | |
| R. Ramazani Darya Sary, [65] | | 2006 | | Iran | | Ghazvin | | 2004-2005 | | Cancer Registry | | 2005 | | 12.9 | | _____ | | 11.08 | | _____ | | 62 | |
| R. Ramazani Darya Sary, [65] | | 2006 | | Iran | | Gilan | | 2004-2005 | | Cancer Registry | | 2005 | | 14.1 | | _____ | | 13.68 | | _____ | | 165 | |
| R. Ramazani Darya Sary, [65] | | 2006 | | Iran | | Golestan | | 2004-2005 | | Cancer Registry | | 2005 | | 10.2 | | _____ | | 8.24 | | _____ | | 67 | |
| R. Ramazani Darya Sary, [65] | | 2006 | | Iran | | Hamadan | | 2004-2005 | | Cancer Registry | | 2005 | | 7.1 | | _____ | | 6.38 | | _____ | | 54 | |
| R. Ramazani Darya Sary, [65] | | 2006 | | Iran | | Hormozgan | | 2004-2005 | | Cancer Registry | | 2005 | | 4.3 | | _____ | | 6.04 | | _____ | | 41 | |
| R. Ramazani Darya Sary, [65] | | 2006 | | Iran | | Ilam | | 2004-2005 | | Cancer Registry | | 2005 | | 7.2 | | _____ | | 4.49 | | _____ | | 12 | |
| R. Ramazani Darya Sary, [65] | | 2006 | | Iran | | Kerman | | 2004-2005 | | Cancer Registry | | 2005 | | 12.6 | | _____ | | 9.24 | | _____ | | 120 | |
| R. Ramazani Darya Sary, [65] | | 2006 | | Iran | | Kermanshah | | 2004-2005 | | Cancer Registry | | 2005 | | 15.5 | | _____ | | 11.6 | | _____ | | 107 | |
| R. Ramazani Darya Sary, [65] | | 2006 | | Iran | | Khorasan rezavi | | 2004-2005 | | Cancer Registry | | 2005 | | 19.6 | | _____ | | 15.34 | | _____ | | 427 | |
| R. Ramazani Darya Sary, [65] | | 2006 | | Iran | | Khozestan | | 2004-2005 | | Cancer Registry | | 2005 | | 15.6 | | _____ | | 10.29 | | _____ | | 215 | |
| R. Ramazani Darya Sary, [65] | | 2006 | | Iran | | Kohkilie boir ahmad | | 2004-2005 | | Cancer Registry | | 2005 | | 4.1 | | _____ | | 4.47 | | _____ | | 14 | |
| R. Ramazani Darya Sary, [65] | | 2006 | | Iran | | Kordestan | | 2004-2005 | | Cancer Registry | | 2005 | | 6.6 | | _____ | | 6.05 | | _____ | | 43 | |
| R. Ramazani Darya Sary, [65] | | 2006 | | Iran | | Lorestan | | 2004-2005 | | Cancer Registry | | 2005 | | 10.01 | | _____ | | 8.78 | | _____ | | 74 | |
| R. Ramazani Darya Sary, [65] | | 2006 | | Iran | | Markazi | | 2004-2005 | | Cancer Registry | | 2005 | | 9.9 | | _____ | | 8.67 | | _____ | | 58 | |
| R. Ramazani Darya Sary, [65] | | 2006 | | Iran | | Mazandaran | | 2004-2005 | | Cancer Registry | | 2005 | | 14.9 | | _____ | | 14.02 | | _____ | | 204 | |
| R. Ramazani Darya Sary, [65] | | 2006 | | Iran | | Qom | | 2004-2005 | | Cancer Registry | | 2005 | | 30.64 | | _____ | | 11.18 | | _____ | | 57 | |
| R. Ramazani Darya Sary, [65] | | 2006 | | Iran | | semnan | | 2004-2005 | | Cancer Registry | | 2005 | | 14.63 | | _____ | | 14.62 | | _____ | | 42 | |
| R. Ramazani Darya Sary, [65] | | 2006 | | Iran | | Sistan and Baluchestan | | 2004-2005 | | Cancer Registry | | 2005 | | 1.5 | | _____ | | 1.69 | | _____ | | 20 | |
| R. Ramazani Darya Sary, [65] | | 2006 | | Iran | | Tehran | | 2004-2005 | | Cancer Registry | | 2005 | | 38.4 | | _____ | | 13.72 | | _____ | | 895 | |
| R. Ramazani Darya Sary, [65] | | 2006 | | Iran | | Yazd | | 2004-2005 | | Cancer Registry | | 2005 | | 25.5 | | _____ | | 18.37 | | _____ | | 87 | |
| R. Ramazani Darya Sary, [65] | | 2006 | | Iran | | zanjan | | 2004-2005 | | Cancer Registry | | 2005 | | 4.9 | | _____ | | 4.14 | | _____ | | 20 | |
| SM. Moosavi, [60] | | 2007 | | Iran | | Ardabil | | 2005-2006 | | Cancer Registry | | 2006 | | 10.3 | | _____ | | 6.88 | | _____ | | 42 | |
| SM. Moosavi, [60] | | 2007 | | Iran | | Azarbaijan gharbi | | 2005-2006 | | Cancer Registry | | 2006 | | 12.8 | | _____ | | 10.11 | | _____ | | 143 | |
| SM. Moosavi, [60] | | 2007 | | Iran | | Azarbaijan sharghi | | 2005-2006 | | Cancer Registry | | 2006 | | 4.7 | | _____ | | 6.52 | | _____ | | 115 | |
| SM. Moosavi, [60] | | 2007 | | Iran | | Booshehr | | 2005-2006 | | Cancer Registry | | 2006 | | 18.4 | | _____ | | 13.39 | | _____ | | 56 | |
| SM. Moosavi, [60] | | 2007 | | Iran | | Chaharmahal bakhtiari | | 2005-2006 | | Cancer Registry | | 2006 | | 11.9 | | _____ | | 7.27 | | _____ | | 31 | |
| **Author** | | **Year of publish** **Data** | | **Country** | | **City** | | **Duration of study** | | **Source of Data** | | **Year of Data** | | **ASR** | | **SE ASR** | | **ICR** | | **SE ICR** | | **Total Breast cases** |  |
| SM. Moosavi, [60] | | 2007 | | Iran | | Fars | | 2005-2006 | | Cancer Registry | | 2006 | | 23.2 | | _____ | | 17.59 | | _____ | | 375 |  |
| SM. Moosavi, [60] | | 2007 | | Iran | | Ghazvin | | 2005-2006 | | Cancer Registry | | 2006 | | 17.3 | | _____ | | 11.08 | | _____ | | 62 |  |
| SM. Moosavi, [60] | | 2007 | | Iran | | Gilan | | 2005-2006 | | Cancer Registry | | 2006 | | 28.4 | | _____ | | 18.49 | | _____ | | 223 |  |
| SM. Moosavi, [60] | | 2007 | | Iran | | Golestan | | 2005-2006 | | Cancer Registry | | 2006 | | 17.5 | | _____ | | 12.79 | | _____ | | 104 |  |
| SM. Moosavi, [60] | | 2007 | | Iran | | Hamadan | | 2005-2006 | | Cancer Registry | | 2006 | | 14.8 | | _____ | | 11.7 | | _____ | | 99 |  |
| SM. Moosavi, [60] | | 2007 | | Iran | | Hormozgan | | 2005-2006 | | Cancer Registry | | 2006 | | 9.7 | | _____ | | 6.93 | | _____ | | 47 |  |
| SM. Moosavi, [60] | | 2007 | | Iran | | Ilam | | 2005-2006 | | Cancer Registry | | 2006 | | 11.7 | | _____ | | 5.61 | | _____ | | 15 |  |
| SM. Moosavi, [60] | | 2007 | | Iran | | Kerman | | 2005-2006 | | Cancer Registry | | 2006 | | 12.2 | | _____ | | 9.16 | | _____ | | 119 |  |
| SM. Moosavi, [60] | | 2007 | | Iran | | Kermanshah | | 2005-2006 | | Cancer Registry | | 2006 | | 14.7 | | _____ | | 11.71 | | _____ | | 108 |  |
| SM. Moosavi, [60] | | 2007 | | Iran | | Khorasan jonobi | | 2005-2006 | | Cancer Registry | | 2006 | | 11.3 | | _____ | | 6.68 | | _____ | | 21 |  |
| SM. Moosavi, [60] | | 2007 | | Iran | | Khorasan rezavi | | 2005-2006 | | Cancer Registry | | 2006 | | 15.2 | | _____ | | 13.22 | | _____ | | 368 |  |
| SM. Moosavi, [60] | | 2007 | | Iran | | Khorasan Shomali | | 2005-2006 | | Cancer Registry | | 2006 | | 9.2 | | _____ | | 6.35 | | _____ | | 26 |  |
| SM. Moosavi, [60] | | 2007 | | Iran | | Khozestan | | 2005-2006 | | Cancer Registry | | 2006 | | 19.9 | | _____ | | 13.73 | | _____ | | 287 |  |
| SM. Moosavi, [60] | | 2007 | | Iran | | Kohkilie boir ahmad | | 2005-2006 | | Cancer Registry | | 2006 | | 5.6 | | _____ | | 3.19 | | _____ | | 10 |  |
| SM. Moosavi, [60] | | 2007 | | Iran | | Kordestan | | 2005-2006 | | Cancer Registry | | 2006 | | 12.4 | | _____ | | 7.04 | | _____ | | 50 |  |
| SM. Moosavi, [60] | | 2007 | | Iran | | Lorestan | | 2005-2006 | | Cancer Registry | | 2006 | | 13.9 | | _____ | | 10.44 | | _____ | | 88 |  |
| SM. Moosavi, [60] | | 2007 | | Iran | | Markazi | | 2005-2006 | | Cancer Registry | | 2006 | | 13.5 | | _____ | | 9.42 | | _____ | | 63 |  |
| SM. Moosavi, [60] | | 2007 | | Iran | | Mazandaran | | 2005-2006 | | Cancer Registry | | 2006 | | 25.3 | | _____ | | 16.83 | | _____ | | 245 |  |
| SM. Moosavi, [60] | | 2007 | | Iran | | Qom | | 2005-2006 | | Cancer Registry | | 2006 | | 14.6 | | _____ | | 11.18 | | _____ | | 57 |  |
| SM. Moosavi, [60] | | 2007 | | Iran | | semnan | | 2005-2006 | | Cancer Registry | | 2006 | | 14.7 | | _____ | | 11.49 | | _____ | | 33 |  |
| SM. Moosavi, [60] | | 2007 | | Iran | | Sistan and Baluchestan | | 2005-2006 | | Cancer Registry | | 2006 | | 5.9 | | _____ | | 2.95 | | _____ | | 35 |  |
| SM. Moosavi, [60] | | 2007 | | Iran | | Tehran | | 2005-2006 | | Cancer Registry | | 2006 | | 27.5 | | _____ | | 17.88 | | _____ | | 1166 |  |
| SM. Moosavi, [60] | | 2007 | | Iran | | Yazd | | 2005-2006 | | Cancer Registry | | 2006 | | 28.1 | | _____ | | 17.73 | | _____ | | 84 |  |
| SM. Moosavi, [60] | | 2007 | | Iran | | zanjan | | 2005-2006 | | Cancer Registry | | 2006 | | 11.6 | | _____ | | 9.11 | | _____ | | 44 |  |
| M, Hagh Azali, [47] | | 2008 | | Iran | | Ardabil | | 2006-2007 | | Cancer Registry | | 2007 | | 25.1 | | _____ | | 18.9 | | _____ | | 6456 |  |
| M, Hagh Azali, [47] | | 2008 | | Iran | | Azarbaijan gharbi | | 2006-2007 | | Cancer Registry | | 2007 | | 12.8 | | _____ | | 10.02 | | _____ | | 139 |  |
| M, Hagh Azali, [47] | | 2008 | | Iran | | Azarbaijan sharghi | | 2006-2007 | | Cancer Registry | | 2007 | | 5.2 | | _____ | | 3.91 | | _____ | | 69 |  |
| M, Hagh Azali, [47] | | 2008 | | Iran | | Booshehr | | 2006-2007 | | Cancer Registry | | 2007 | | 22.1 | | _____ | | 17.22 | | _____ | | 72 |  |
| M, Hagh Azali, [47] | | 2008 | | Iran | | Chaharmahal bakhtiari | | 2006-2007 | | Cancer Registry | | 2007 | | 11.3 | | _____ | | 8.68 | | _____ | | 37 |  |
| M, Hagh Azali, [47] | | 2008 | | Iran | | Isfahan | | 2006-2007 | | Cancer Registry | | 2007 | | 30.4 | | _____ | | 22.53 | | _____ | | 501 |  |

| **Author** | **Year of publish** **Data** | **Country** | **City** | **Duration of study** | **Source of Data** | **Year of Data** | **ASR** | **SE ASR** | **ICR** | **SE ICR** | **Total Breast cases** |
| --- | --- | --- | --- | --- | --- | --- | --- | --- | --- | --- | --- |
| M, Hagh Azali, [47] | 2008 | Iran | Fars | 2006-2007 | Cancer Registry | 2007 | 23.4 | _____ | 17.5 | _____ | 373 |
| M, Hagh Azali, [47] | 2008 | Iran | Ghazvin | 2006-2007 | Cancer Registry | 2007 | 18.7 | _____ | 14.5 | _____ | 80 |
| M, Hagh Azali, [47] | 2008 | Iran | Gilan | 2006-2007 | Cancer Registry | 2007 | 25.4 | _____ | 18.15 | _____ | 219 |
| M, Hagh Azali, [47] | 2008 | Iran | Golestan | 2006-2007 | Cancer Registry | 2007 | 19.8 | _____ | 14.64 | _____ | 119 |
| M, Hagh Azali, [47] | 2008 | Iran | Hamadan | 2006-2007 | Cancer Registry | 2007 | 19.1 | _____ | 14.18 | _____ | 120 |
| M, Hagh Azali, [47] | 2008 | Iran | Hormozgan | 2006-2007 | Cancer Registry | 2007 | 15.2 | _____ | 11.5 | _____ | 78 |
| M, Hagh Azali, [47] | 2008 | Iran | Ilam | 2006-2007 | Cancer Registry | 2007 | 13.1 | _____ | 10.95 | _____ | 29 |
| M, Hagh Azali, [47] | 2008 | Iran | Kerman | 2006-2007 | Cancer Registry | 2007 | 18.03 | _____ | 13.4 | _____ | 174 |
| M, Hagh Azali, [47] | 2008 | Iran | Kermanshah | 2006-2007 | Cancer Registry | 2007 | 16.5 | _____ | 13.1 | _____ | 119 |
| M, Hagh Azali, [47] | 2008 | Iran | Khorasan jonobi | 2006-2007 | Cancer Registry | 2007 | 12.7 | _____ | 7.95 | _____ | 25 |
| M, Hagh Azali, [47] | 2008 | Iran | Khorasan rezavi | 2006-2007 | Cancer Registry | 2007 | 23.1 | _____ | 17.28 | _____ | 481 |
| M, Hagh Azali, [47] | 2008 | Iran | Khorasan Shomali | 2006-2007 | Cancer Registry | 2007 | 6.5 | _____ | 4.64 | _____ | 19 |
| M, Hagh Azali, [47] | 2008 | Iran | Khozestan | 2006-2007 | Cancer Registry | 2007 | 23.5 | _____ | 17.51 | _____ | 366 |
| M, Hagh Azali, [47] | 2008 | Iran | Kohkilie boir ahmad | 2006-2007 | Cancer Registry | 2007 | 7.8 | _____ | 5.74 | _____ | 18 |
| M, Hagh Azali, [47] | 2008 | Iran | Kordestan | 2006-2007 | Cancer Registry | 2007 | 12.8 | _____ | 9.15 | _____ | 65 |
| M, Hagh Azali, [47] | 2008 | Iran | Lorestan | 2006-2007 | Cancer Registry | 2007 | 13.9 | _____ | 10.44 | _____ | 88 |
| M, Hagh Azali, [47] | 2008 | Iran | Markazi | 2006-2007 | Cancer Registry | 2007 | 18.8 | _____ | 13.75 | _____ | 92 |
| M, Hagh Azali, [47] | 2008 | Iran | Mazandaran | 2006-2007 | Cancer Registry | 2007 | 23.7 | _____ | 17.31 | _____ | 252 |
| M, Hagh Azali, [47] | 2008 | Iran | Qom | 2006-2007 | Cancer Registry | 2007 | 20.4 | _____ | 14.91 | _____ | 76 |
| M, Hagh Azali, [47] | 2008 | Iran | semnan | 2006-2007 | Cancer Registry | 2007 | 19.8 | _____ | 14.62 | _____ | 42 |
| M, Hagh Azali, [47] | 2008 | Iran | Sistan and Baluchestan | 2006-2007 | Cancer Registry | 2007 | 5.9 | _____ | 4.56 | _____ | 54 |
| M, Hagh Azali, [47] | 2008 | Iran | Tehran | 2006-2007 | Cancer Registry | 2007 | 32.1 | _____ | 22.96 | _____ | 1497 |
| M, Hagh Azali, [47] | 2008 | Iran | Yazd | 2006-2007 | Cancer Registry | 2007 | 31.98 | _____ | 23.01 | _____ | 109 |
| M, Hagh Azali, [47] | 2008 | Iran | zanjan | 2006-2007 | Cancer Registry | 2007 | 7.9 | _____ | 6.01 | _____ | 29 |
| K. Etemad, [37] | 2010 | Iran | Ardabil | 2007-2008 | Cancer Registry | 2008 | 8.3 | _____ | 6.38 | _____ | 39 |
| K. Etemad, [37] | 2010 | Iran | Azarbaijan gharbi | 2007-2008 | Cancer Registry | 2008 | 19.8 | _____ | 14.63 | _____ | 207 |
| K. Etemad, [37] | 2010 | Iran | Azarbaijan sharghi | 2007-2008 | Cancer Registry | 2008 | 22.8 | _____ | 17.52 | _____ | 309 |
| K. Etemad, [37] | 2010 | Iran | Booshehr | 2007-2008 | Cancer Registry | 2008 | 24.8 | _____ | 19.37 | _____ | 81 |
| K. Etemad, [37] | 2010 | Iran | Chaharmahal bakhtiari | 2007-2008 | Cancer Registry | 2008 | 11.4 | _____ | 8.45 | _____ | 36 |
| K. Etemad, [37] | 2010 | Iran | Isfahan | 2007-2008 | Cancer Registry | 2008 | 33.3 | _____ | 24.82 | _____ | 552 |

| **Author** | **Year of publish** **Data** | **Country** | **City** | **Duration of study** | **Source of Data** | **Year of Data** | **ASR** | **SE ASR** | **ICR** | **SE ICR** | **Total Breast cases** |
| --- | --- | --- | --- | --- | --- | --- | --- | --- | --- | --- | --- |
| K. Etemad, [37] | 2010 | Iran | Fars | 2007-2008 | Cancer Registry | 2008 | 29.2 | _____ | 21.25 | _____ | 453 |
| K. Etemad, [37] | 2010 | Iran | Ghazvin | 2007-2008 | Cancer Registry | 2008 | 19.1 | _____ | 13.95 | _____ | 78 |
| K. Etemad, [37] | 2010 | Iran | Gilan | 2007-2008 | Cancer Registry | 2008 | 29.5 | _____ | 20.81 | _____ | 251 |
| K. Etemad, [37] | 2010 | Iran | Golestan | 2007-2008 | Cancer Registry | 2008 | 17.6 | _____ | 13.41 | _____ | 109 |
| K. Etemad, [37] | 2010 | Iran | Hamadan | 2007-2008 | Cancer Registry | 2008 | 17.8 | _____ | 13.11 | _____ | 111 |
| K. Etemad, [37] | 2010 | Iran | Hormozgan | 2007-2008 | Cancer Registry | 2008 | 12.8 | _____ | 9.88 | _____ | 67 |
| K. Etemad, [37] | 2010 | Iran | Ilam | 2007-2008 | Cancer Registry | 2008 | 14.5 | _____ | 11.6 | _____ | 31 |
| K. Etemad, [37] | 2010 | Iran | Kerman | 2007-2008 | Cancer Registry | 2008 | 15.8 | _____ | 11.63 | _____ | 151 |
| K. Etemad, [37] | 2010 | Iran | Kermanshah | 2007-2008 | Cancer Registry | 2008 | 26.9 | _____ | 20.17 | _____ | 186 |
| K. Etemad, [37] | 2010 | Iran | Khorasan jonobi | 2007-2008 | Cancer Registry | 2008 | 15.6 | _____ | 11.4 | _____ | 31 |
| K. Etemad, [37] | 2010 | Iran | Khorasan rezavi | 2007-2008 | Cancer Registry | 2008 | 27.3 | _____ | 20.37 | _____ | 567 |
| K. Etemad, [37] | 2010 | Iran | Khorasan Shomali | 2007-2008 | Cancer Registry | 2008 | 8.8 | _____ | 6.1 | _____ | 25 |
| K. Etemad, [37] | 2010 | Iran | Khozestan | 2007-2008 | Cancer Registry | 2008 | 26.1 | _____ | 19.86 | _____ | 415 |
| K. Etemad, [37] | 2010 | Iran | Kohkilie boir ahmad | 2007-2008 | Cancer Registry | 2008 | 6.03 | _____ | 4.79 | _____ | 15 |
| K. Etemad, [37] | 2010 | Iran | Kordestan | 2007-2008 | Cancer Registry | 2008 | 15.5 | _____ | 11.26 | _____ | 80 |
| K. Etemad, [37] | 2010 | Iran | Lorestan | 2007-2008 | Cancer Registry | 2008 | 17.8 | _____ | 13.05 | _____ | 110 |
| K. Etemad, [37] | 2010 | Iran | Markazi | 2007-2008 | Cancer Registry | 2008 | 19.8 | _____ | 14.5 | _____ | 97 |
| K. Etemad, [37] | 2010 | Iran | Mazandaran | 2007-2008 | Cancer Registry | 2008 | 24.9 | _____ | 18.34 | _____ | 267 |
| K. Etemad, [37] | 2010 | Iran | Qom | 2007-2008 | Cancer Registry | 2008 | 24.4 | _____ | 18.24 | _____ | 93 |
| K. Etemad, [37] | 2010 | Iran | semnan | 2007-2008 | Cancer Registry | 2008 | 20.4 | _____ | 14.27 | _____ | 41 |
| K. Etemad, [37] | 2010 | Iran | Sistan and Baluchestan | 2007-2008 | Cancer Registry | 2008 | 5.7 | _____ | 4.56 | _____ | 54 |
| K. Etemad, [37] | 2010 | Iran | Tehran | 2007-2008 | Cancer Registry | 2008 | 33.9 | _____ | 24.31 | _____ | 1585 |
| K. Etemad, [37] | 2010 | Iran | Yazd | 2007-2008 | Cancer Registry | 2008 | 25.6 | _____ | 19 | _____ | 90 |
| K. Etemad, [37] | 2010 | Iran | zanjan | 2007-2008 | Cancer Registry | 2008 | 13.6 | _____ | 10.35 | _____ | 50 |
| K. Etemad, [38] | 2011 | Iran | Ardabil | 2008-2009 | Cancer Registry | 2009 | 9.7 | _____ | 7.37 | _____ | 45 |
| K. Etemad, [38] | 2011 | Iran | Azarbaijan gharbi | 2008-2009 | Cancer Registry | 2009 | 13.5 | _____ | 10.25 | _____ | 145 |
| K. Etemad, [38] | 2011 | Iran | Azarbaijan sharghi | 2008-2009 | Cancer Registry | 2009 | 34.1 | _____ | 25.62 | _____ | 452 |
| K. Etemad, [38] | 2011 | Iran | Booshehr | 2008-2009 | Cancer Registry | 2009 | 26.97 | _____ | 20.8 | _____ | 87 |
| K. Etemad, [38] | 2011 | Iran | Chaharmahal bakhtiari | 2008-2009 | Cancer Registry | 2009 | 18.7 | _____ | 14.08 | _____ | 60 |
| K. Etemad, [38] | 2011 | Iran | Isfahan | 2008-2009 | Cancer Registry | 2009 | 37.3 | _____ | 27.29 | _____ | 607 |

| **Author** | **Year of publish** | **Country** | **City** | **Duration of study** | **Source of Data** | **Year of Data** | **ASR** | **SE ASR** | **ICR** | **SE ICR** | **Total Breast cases** |
| --- | --- | --- | --- | --- | --- | --- | --- | --- | --- | --- | --- |
| K. Etemad, [38] | 2011 | Iran | Fars | 2008-2009 | Cancer Registry | 2009 | 32.3 | _____ | 22.94 | _____ | 489 |
| K. Etemad, [38] | 2011 | Iran | Ghazvin | 2008-2009 | Cancer Registry | 2009 | 27.8 | _____ | 20.56 | _____ | 115 |
| K. Etemad, [38] | 2011 | Iran | Gilan | 2008-2009 | Cancer Registry | 2009 | 32.7 | _____ | 22.63 | _____ | 273 |
| K. Etemad, [38] | 2011 | Iran | Golestan | 2008-2009 | Cancer Registry | 2009 | 20.4 | _____ | 15.01 | _____ | 122 |
| K. Etemad, [38] | 2011 | Iran | Hamadan | 2008-2009 | Cancer Registry | 2009 | 24.6 | _____ | 18.08 | _____ | 153 |
| K. Etemad, [38] | 2011 | Iran | Hormozgan | 2008-2009 | Cancer Registry | 2009 | 18.6 | _____ | 14 | _____ | 95 |
| K. Etemad, [38] | 2011 | Iran | Ilam | 2008-2009 | Cancer Registry | 2009 | 12.1 | _____ | 9.36 | _____ | 25 |
| K. Etemad, [38] | 2011 | Iran | Kerman | 2008-2009 | Cancer Registry | 2009 | 16.4 | _____ | 12.32 | _____ | 160 |
| K. Etemad, [38] | 2011 | Iran | Kermanshah | 2008-2009 | Cancer Registry | 2009 | 27.2 | _____ | 20.93 | _____ | 193 |
| K. Etemad, [38] | 2011 | Iran | Khorasan jonobi | 2008-2009 | Cancer Registry | 2009 | 20.5 | _____ | 13.05 | _____ | 41 |
| K. Etemad, [38] | 2011 | Iran | Khorasan rezavi | 2008-2009 | Cancer Registry | 2009 | 28.8 | _____ | 21.48 | _____ | 598 |
| K. Etemad, [38] | 2011 | Iran | Khorasan Shomali | 2008-2009 | Cancer Registry | 2009 | 15.8 | _____ | 12.2 | _____ | 50 |
| K. Etemad, [38] | 2011 | Iran | Khozestan | 2008-2009 | Cancer Registry | 2009 | 41 | _____ | 30.76 | _____ | 643 |
| K. Etemad, [38] | 2011 | Iran | Kohkilie boir ahmad | 2008-2009 | Cancer Registry | 2009 | 7.2 | _____ | 6.06 | _____ | 19 |
| K. Etemad, [38] | 2011 | Iran | Kordestan | 2008-2009 | Cancer Registry | 2009 | 21.2 | _____ | 16.47 | _____ | 117 |
| K. Etemad, [38] | 2011 | Iran | Lorestan | 2008-2009 | Cancer Registry | 2009 | 19.8 | _____ | 14.83 | _____ | 125 |
| K. Etemad, [38] | 2011 | Iran | Markazi | 2008-2009 | Cancer Registry | 2009 | 19.9 | _____ | 14.35 | _____ | 96 |
| K. Etemad, [38] | 2011 | Iran | Mazandaran | 2008-2009 | Cancer Registry | 2009 | 39.8 | _____ | 29.06 | _____ | 423 |
| K. Etemad, [38] | 2011 | Iran | Qom | 2008-2009 | Cancer Registry | 2009 | 24.8 | _____ | 19.22 | _____ | 98 |
| K. Etemad, [38] | 2011 | Iran | semnan | 2008-2009 | Cancer Registry | 2009 | 41.5 | _____ | 30.63 | _____ | 88 |
| K. Etemad, [38] | 2011 | Iran | Sistan and Baluchestan | 2008-2009 | Cancer Registry | 2009 | 5.7 | _____ | 4.47 | _____ | 53 |
| K. Etemad, [38] | 2011 | Iran | Tehran | 2008-2009 | Cancer Registry | 2009 | 55.9 | _____ | 40.24 | _____ | 2624 |
| K. Etemad, [38] | 2011 | Iran | Yazd | 2008-2009 | Cancer Registry | 2009 | 31.4 | _____ | 23.64 | _____ | 112 |
| K. Etemad, [38] | 2011 | Iran | zanjan | 2008-2009 | Cancer Registry | 2009 | 10.2 | _____ | 7.66 | _____ | 37 |
| K. Etemad, [39] | 2012 | Iran | Ardabil | 2009-2010 | Cancer Registry | 2010 | 22.47 | _____ | 10.31 | _____ | 63 |
| K. Etemad, [39] | 2012 | Iran | Azarbaijan gharbi | 2009-2010 | Cancer Registry | 2010 | 28.9 | _____ | 12.58 | _____ | 178 |
| K. Etemad, [39] | 2012 | Iran | Azarbaijan sharghi | 2009-2010 | Cancer Registry | 2010 | 61.48 | _____ | 23.01 | _____ | 406 |
| K. Etemad, [39] | 2012 | Iran | Booshehr | 2009-2010 | Cancer Registry | 2010 | 51.97 | _____ | 21.76 | _____ | 91 |
| K. Etemad, [39] | 2012 | Iran | Chaharmahal bakhtiari | 2009-2010 | Cancer Registry | 2010 | 37.48 | _____ | 13.61 | _____ | 68 |
| K. Etemad, [39] | 2012 | Iran | Isfahan | 2009-2010 | Cancer Registry | 2010 | 75.6 | _____ | 28.91 | _____ | 643 |

| **Author** | **Year of publish** | **Country** | **City** | **Duration of study** | **Source of Data** | **Year of Data** | **ASR** | **SE ASR** | **ICR** | **SE ICR** | **Total Breast cases** |
| --- | --- | --- | --- | --- | --- | --- | --- | --- | --- | --- | --- |
| K. Etemad, [39] | 2012 | Iran | Fars | 2009-2010 | Cancer Registry | 2010 | 65.6 | _____ | 28.1 | _____ | 599 |
| K. Etemad, [39] | 2012 | Iran | Ghazvin | 2009-2010 | Cancer Registry | 2010 | 42.5 | _____ | 15.2 | _____ | 85 |
| K. Etemad, [39] | 2012 | Iran | Gilan | 2009-2010 | Cancer Registry | 2010 | 55.97 | _____ | 21.88 | _____ | 264 |
| K. Etemad, [39] | 2012 | Iran | Golestan | 2009-2010 | Cancer Registry | 2010 | 35.5 | _____ | 15.38 | _____ | 125 |
| K. Etemad, [39] | 2012 | Iran | Hamadan | 2009-2010 | Cancer Registry | 2010 | 47.1 | _____ | 17.49 | _____ | 148 |
| K. Etemad, [39] | 2012 | Iran | Hormozgan | 2009-2010 | Cancer Registry | 2010 | 29.3 | _____ | 9.43 | _____ | 64 |
| K. Etemad, [39] | 2012 | Iran | Ilam | 2009-2010 | Cancer Registry | 2010 | 24.72 | _____ | 10.1 | _____ | 27 |
| K. Etemad, [39] | 2012 | Iran | Kerman | 2009-2010 | Cancer Registry | 2010 | 38.6 | _____ | 18.41 | _____ | 239 |
| K. Etemad, [39] | 2012 | Iran | Khorasan jonobi | 2009-2010 | Cancer Registry | 2010 | 27.61 | _____ | 10.18 | _____ | 32 |
| K. Etemad, [39] | 2012 | Iran | Khorasan rezavi | 2009-2010 | Cancer Registry | 2010 | 49.8 | _____ | 18.97 | _____ | 528 |
| K. Etemad, [39] | 2012 | Iran | Khorasan Shomali | 2009-2010 | Cancer Registry | 2010 | 25.2 | _____ | 10.01 | _____ | 41 |
| K. Etemad, [39] | 2012 | Iran | Khozestan | 2009-2010 | Cancer Registry | 2010 | 70.85 | _____ | 0 | _____ | 525 |
| K. Etemad, [39] | 2012 | Iran | Kohkilie boir ahmad | 2009-2010 | Cancer Registry | 2010 | 21.8 | _____ | 11.81 | _____ | 37 |
| K. Etemad, [39] | 2012 | Iran | Kordestan | 2009-2010 | Cancer Registry | 2010 | 38.6 | _____ | 15.2 | _____ | 100 |
| K. Etemad, [39] | 2012 | Iran | Lorestan | 2009-2010 | Cancer Registry | 2010 | 39.02 | _____ | 14.95 | _____ | 126 |
| K. Etemad, [39] | 2012 | Iran | Markazi | 2009-2010 | Cancer Registry | 2010 | 54.2 | _____ | 28.85 | _____ | 193 |
| K. Etemad, [39] | 2012 | Iran | Mazandaran | 2009-2010 | Cancer Registry | 2010 | 62.6 | _____ | 25.14 | _____ | 356 |
| K. Etemad, [39] | 2012 | Iran | Qom | 2009-2010 | Cancer Registry | 2010 | 34.84 | _____ | 10.59 | _____ | 54 |
| K. Etemad, [39] | 2012 | Iran | semnan | 2009-2010 | Cancer Registry | 2010 | 68.7 | _____ | 20.54 | _____ | 59 |
| K. Etemad, [39] | 2012 | Iran | Sistan and Baluchestan | 2009-2010 | Cancer Registry | 2010 | 10.2 | _____ | 4.9 | _____ | 58 |
| K. Etemad, [39] | 2012 | Iran | Tehran | 2009-2010 | Cancer Registry | 2010 | 93.3 | _____ | 32.02 | _____ | 2088 |
| K. Etemad, [39] | 2012 | Iran | Yazd | 2009-2010 | Cancer Registry | 2010 | 73.2 | _____ | 28.5 | _____ | 135 |
| K. Etemad, [39] | 2012 | Iran | zanjan | 2009-2010 | Cancer Registry | 2010 | 20.6 | _____ | 9.53 | _____ | 46 |
| K. Etemad, [39] | 2012 | Iran | Kermanshah | 2009-2010 | Cancer Registry | 2010 | 52 | _____ | 0 | _____ | 164 |
| [19] | 2013 | Iran | Iran | 2010-2011 | Cancer Registry | 2011 | 30.2 | _____ | 23.5 | _____ | 8069 |
| K. Etemad, [39] | 2012 | Iran | Iran | 2009-2010 | Cancer Registry | 2010 | 28.2 | _____ | 22.1 | _____ | 7582 |
| K. Etemad, [39] | 2012 | Iran | Iran | 2008-2009 | Cancer Registry | 2009 | 33.21 | _____ | 24.9 | _____ | 8424 |
| K. Etemad, [39] | 2012 | Iran | Iran | 2007-2008 | Cancer Registry | 2008 | 33.2 | _____ | 20.4 | _____ | 6976 |
| K. Etemad, [39] | 2012 | Iran | Iran | 2006-2007 | Cancer Registry | 2007 | 27.1 | _____ | 18.9 | _____ | 6456 |
| K. Etemad, [39] | 2012 | Iran | Iran | 2005-2006 | Cancer Registry | 2006 | 25.1 | _____ | 17.4 | _____ | 5981 |
| K. Etemad, [39] | 2012 | Iran | Iran | 2004-2005 | Cancer Registry | 2005 | 23.2 | _____ | 13.8 | _____ | 4557 |
| K. Etemad, [39] | 2012 | Iran | Iran | 2003-2004 | Cancer Registry | 2004 | 15.9 | _____ | 12.2 | _____ | 3946 |

| Author | **Year of publish** | **Country** | **City** | **Duration of study** | **Source of Data** | **Year of Data** | **ASR** | **SE ASR** | **ICR** | **SE ICR** | **Total Breast cases** |
| --- | --- | --- | --- | --- | --- | --- | --- | --- | --- | --- | --- |
| M. A. Mashhadi,[56] | 2010 | Iran | Fars | 1998-2002 | Cancer Registry | 2000 | 13 | _____ | 8.6 | _____ | 877 |
| M. A. Mohagheghi, [59] | 2009 | Iran | Tehran | 1998-2001 | Cancer Registry | 2000 | 31.4 | _____ | 24.8 | _____ | 3579 |
| M. A. N. Al-Madouj,[21] | 2011 | Kuwait | Kuwait | 1998-2002 | GCC | 2000 | 45.3 | _____ | _____ | _____ | 562 |
| M. A. N. Al-Madouj,[21] | 2011 | Kuwait | Kuwait | 2003-2008 | GCC | 2005 | 50 | _____ | _____ | _____ | 788 |
| M. A. N. Al-Madouj,[21] | 2011 | Saudi Arabia | Saudi Arabia | 1998-2002 | GCC | 2000 | 13.6 | _____ | _____ | _____ | 2903 |
| M. A. N. Al-Madouj,[21] | 2011 | Saudi Arabia | Saudi Arabia | 2003-2007 | GCC | 2005 | 18.5 | _____ | _____ | _____ | 4734 |
| M. A. N. Al-Madouj,[21] | 2011 | Oman | Oman | 1998-2002 | GCC | 2000 | 14.3 | _____ | _____ | _____ | 332 |
| M. A. N. Al-Madouj,[21] | 2011 | Oman | Oman | 2003-2007 | GCC | 2005 | 20.6 | _____ | _____ | _____ | 439 |
| M. A. N. Al-Madouj,[21] | 2011 | UAE | UAE | 1998-2002 | GCC | 2000 | 21.6 | _____ | _____ | _____ | 194 |
| M. A. N. Al-Madouj,[21] | 2011 | UAE | UAE | 2003-2007 | GCC | 2000 | 28.2 | _____ | _____ | _____ | 293 |
| M. A. N. Al-Madouj,[21] | 2011 | Qatar | Qatar | 1998-2002 | GCC | 2000 | 36.6 | _____ | _____ | _____ | 92 |
| M. A. N. Al-Madouj,[21] | 2011 | Qatar | Qatar | 2003-2008 | GCC | 2006 | 53.3 | _____ | _____ | _____ | 149 |
| M. A. N. Al-Madouj,[21] | 2011 | Bahrain | Bahrain | 1998-2002 | GCC | 2000 | 49.4 | _____ | _____ | _____ | 358 |
| M. A. N. Al-Madouj,[21] | 2011 | Bahrain | Bahrain | 2003-2008 | GCC | 2006 | 58.7 | _____ | _____ | _____ | 452 |
| M. A. Qureshi, [64] | 2016 | Pakistan | Karachi | 2010-2015 | Pathology | 2012 | 43.1 | _____ | 57.4 | _____ | 3889 |
| M. A. Tazi,[74] | 2013 | Morocco | Rabat | 2006-2008 | Cancer Registry | 2007 | 43.4 | _____ | 49.2 | _____ | 491 |
| M. A. TAZI,[74] | 2009 | Morocco | Rabat | 2005 | Cancer Registry | 2005 | 35.8 | _____ | 38.8 | _____ | 127 |
| M. Babaei, [24] | 2005 | Iran | Semnan | 1998-2002 | Cancer Registry | 2000 | 21.3 | _____ | 20.1 | _____ |  |
| M. BEN ABDALLAH, [26] |  | Tunisia | North | 2004-2006 | Cancer Registry | 2005 | 31.84 | _____ | 32.33 | _____ | 2354 |
| M. El Mistiri, [35] | 2007 | Libya | Benghazi | 2003 | Cancer Registry | 2003 | 22.9 | _____ | _____ | _____ | 116 |
| M. El Mistiri,[35] | 2007 | Libya | Benghazi | 2002 | Globo can | 2002 | 17.4 | _____ | _____ | _____ |  |
| M. El Mistiri,[34] | 2015 | Libya | Benghazi | 2003-2005 | Cancer Registry | 2004 | 23 | _____ | _____ | _____ | 365 |

| **Author** | **Year of publish** **Data** | **Country** | **City** | **Duration of study** | **Source of Data** | **Year of Data** | **ASR** | **SE ASR** | **ICR** | **SE ICR** | **Total Breast cases** |
| --- | --- | --- | --- | --- | --- | --- | --- | --- | --- | --- | --- |
| M. Fararouei, [41] | 2015 | Iran | Iran | 2006 | Cancer Registry | 2006 | 23.6 | _____ | _____ | _____ |  |
| M. Fararouei, [41] | 2015 | Iran | Kohkilie Boir Ahmad | 2007-2009 | Cancer Registry | 2008 | 5.3 | _____ | 9.6 | _____ | 30 |
| M. Fateh,[42] | 2013 | Iran | Semnan(Shahroud) | 2000-2010 | Cancer Registry | 2005 | 16.5 | _____ | 14.6 | _____ |  |
| World Health Organization [11] | 2015 | Yemen | Yemen | 2012 | Globo can | 2012 | 27.4 | _____ | 15.5 | _____ | 1963 |
| World Health Organization [11] | 2015 | Oman | Oman | 2012 | Globo can | 2012 | 25.8 | _____ | 16.4 | _____ | 195 |
| World Health Organization [11] | 2015 | Afghanistan | Afghanistan | 2012 | Globo can | 2012 | 35.1 | _____ | 19.3 | _____ | 3108 |
| World Health Organization [11] | 2015 | Saudi Arabia | Saudi Arabia | 2012 | Globo can | 2012 | 29.5 | _____ | 21.7 | _____ | 2791 |
| World Health Organization [11] | 2015 | UAE | UAE | 2012 | Globo can | 2012 | 39.5 | _____ | 22.8 | _____ | 568 |
| World Health Organization [11] | 2015 | Iran | Iran | 2012 | Globo can | 2012 | 28.1 | _____ | 26.3 | _____ | 9795 |
| World Health Organization [11] | 2015 | Kuwait | Kuwait | 2012 | Globo can | 2012 | 46.7 | _____ | 26.9 | _____ | 314 |
| World Health Organization [11] | 2015 | Iraq | Iraq | 2012 | Globo can | 2012 | 42.6 | _____ | 27.1 | _____ | 4542 |
| World Health Organization [11] | 2015 | Qatar | Qatar | 2012 | Globo can | 2012 | 46.1 | _____ | 31.6 | _____ | 148 |
| World Health Organization [11] | 2015 | Bahrain | Bahrain | 2012 | Globo can | 2012 | 42.5 | _____ | 34.7 | _____ | 177 |
| World Health Organization [11] | 2015 | Pakistan | Pakistan | 2012 | Globo can | 2012 | 50.3 | _____ | 38.4 | _____ | 34038 |
| World Health Organization [11] | 2015 | Jordan | Jordan | 2012 | Globo can | 2012 | 61 | _____ | 39.4 | _____ | 1237 |
| World Health Organization [11] | 2015 | Syria | Syria | 2012 | Globo can | 2012 | 52.5 | _____ | 39.7 | _____ | 4140 |
| World Health Organization [11] | 2015 | Lebanon | Lebanon | 2012 | Globo can | 2012 | 78.7 | _____ | 88 | _____ | 1934 |
| M. H. Somi,[71] | 2008 | East Azerbaijan Province | East Azerbaijan Province | 2006-2007 | Cancer Registry | 2007 | 23.5 | _____ | 22.3 | _____ | 393 |
| M. Hanif, [48] | 2015 | Pakistan | Pakistan | 2000-2012 | Hospital | 2006 | 37.13 | _____ | _____ | _____ | 5574 |
| M. Hanif, [49] | 2009 | Pakistan | Pakistan | 2000-2008 | Radiotherapy | 2004 | 38.2 | _____ | _____ | _____ | 3243 |
| M. M. Al-Hashimi [77] | 2014 | Iraq | Iraq | 2000-2009 | Cancer Registry | 2000 | 26.6 | _____ | _____ | _____ | 23792 |
| M. M. Al-Hashimi [77] | 2014 | Iraq | Iraq | 2000-2009 | Cancer Registry | 2009 | 31.5 | _____ | _____ | _____ | 23792 |
| N. A. Lakkis,[81] | 2010 | Lebanon | Lebanon | 2004 | Cancer Registry | 2004 | 71 | _____ | 64.1 | _____ |  |
| N. Khoshnaw,[54] | 2015 | Iraq | Sulaymaniyah | 2006-2014 | Hospital | 2010 | 17.1 | _____ | _____ | _____ |  |

| **Author** | **Year of publish** **Data** | **Country** | **City** | **Duration of study** | **Source of Data** | **Year of Data** | **ASR** | **SE ASR** | **ICR** | **SE ICR** | **Total Breast cases** |
| --- | --- | --- | --- | --- | --- | --- | --- | --- | --- | --- | --- |
| S. B. Jazayeri, [78] | 2015 | Iran | Mashhad | 2000-2010 | Cancer Registry | 2005 | 29.35 | _____ | 25.01 | _____ | _____ |
| S. B. Jazayeri,[78] | 2015 | Iran | Ahvaz | 2000-2010 | Cancer Registry | 2005 | 48.6 | _____ | 35.4 | _____ | _____ |
| S. B. Jazayeri, [78] | 2015 | Iran | Shiraz | 2000-2010 | Cancer Registry | 2005 | 46.5 | _____ | 43.9 | _____ | _____ |
| S. Dey, [33] | 2010 | Egypt | Gharbiah | 1999-2006 | Cancer Registry | 1999 | 31.75 | _____ | 4.63 | _____ | 560 |
| S. Dey, [33] | 2010 | Egypt | Gharbiah | 1999-2006 | Cancer Registry | 2000 | 30.22 | _____ | 4.5 | _____ | 541 |
| S. Dey, [33] | 2010 | Egypt | Gharbiah | 1999-2006 | Cancer Registry | 2001 | 31.81 | _____ | 3.67 | _____ | 577 |
| S. Dey, [33] | 2010 | Egypt | Gharbiah | 1999-2006 | Cancer Registry | 2002 | 33.67 | _____ | 4.42 | _____ | 621 |
| S. Dey, [33] | 2010 | Egypt | Gharbiah | 1999-2006 | Cancer Registry | 2003 | 32.93 | _____ | 2.63 | _____ | 613 |
| S. Dey, [33] | 2010 | Egypt | Gharbiah | 1999-2006 | Cancer Registry | 2004 | 35.11 | _____ | 2.97 | _____ | 665 |
| S. Dey, [33] | 2010 | Egypt | Gharbiah | 1999-2006 | Cancer Registry | 2005 | 31.81 | _____ | 2.83 | _____ | 617 |
| S. Dey, [33] | 2010 | Egypt | Gharbiah | 1999-2006 | Cancer Registry | 2006 | 34.49 | _____ | 2.71 | _____ | 681 |
| S. H. O, A. H. L, [91] | 2016 | Iraq | Basreh | 2005-2012 | Cancer Registry | 2008 | 34.6 | _____ | 24.1 | _____ | 2213 |
| S. Hashemzadeh,[50] | 2012 | Iran | Tabriz | 2003-2008 | Cancer Registry | 2006 | 35.9 | _____ |  | _____ | 868 |
| S. I. Ismail,[53] | 2013 | Jordan | Jordan | 1996-2000 | Cancer Registry | 1998 | 38 | _____ | 21.4 | _____ | 2371 |
| S. I. Ismail, [53] | 2013 | Jordan | Jordan | 1996-2006 | Cancer Registry | 2006 | 48.2 | _____ | 29.1 | _____ | 8536 |
| S. M. Masoompour, [57] | 2016 | Iran | Fars | 2007-2010 | Cancer Registry | 2009 | 28.4 | _____ | 23.35 | _____ | _____ |
| S. M. Mousavi, [62] | 2006 | Iran | Tehran | 1998-2001 | Cancer Registry | 1998 | 18.2 | _____ | _____ | _____ | _____ |
| S. M. Mousavi, [62] | 2006 | Iran | Tehran | 1998-2001 | Cancer Registry | 1999 | 15.9 | _____ | _____ | _____ | _____ |
| S. M. Mousavi, [62] | 2006 | Iran | Tehran | 1998-2001 | Cancer Registry | 2000 | 19.5 | _____ | _____ | _____ | _____ |
| S. M. Mousavi, [62] | 2006 | Iran | Tehran | 1998-2001 | Cancer Registry | 2001 | 14.8 | _____ | _____ | _____ | _____ |
| World Health Organization[11] | 2015 | Morocco | Morocco | 2012 | Globo can | 2012 | 40.8 | _____ | _____ | _____ | 6650 |
| World Health Organization[11] | 2015 | Libya | Libya | 2012 | Globo can | 2012 | 24.1 | _____ | _____ | _____ | 679 |
| World Health Organization[11] | 2015 | Egypt | Egypt | 2012 | Globo can | 2012 | 47.1 | _____ | _____ | _____ | 21945 |
| World Health Organization[11] | 2015 | Tunisia | Tunisia | 2012 | Globo can | 2012 | 31.8 | _____ | _____ | _____ | 1826 |
| World Health Organization[11] | 2015 | Egypt | Egypt | 2012 | Globo can | 2012 | 49.5 | _____ | _____ | _____ | 18660 |

| **Author** | **Year of publish Data** | **Country** | **City** | **Duration of study** | **Source of Data** | **Year of Data** | **ASR** | **SE ASR** | **ICR** | **SE ICR** | **Total Breast cases** |
| --- | --- | --- | --- | --- | --- | --- | --- | --- | --- | --- | --- |
| World Health Organization[11] | 2015 | Sudan | Sudan | 2012 | Globo can | 2012 | 27.8 | _____ | _____ | _____ | 3439 |
| World Health Organization[11] | 2015 | Djibouti | Djibouti | 2012 | Globo can | 2012 | 35.9 | _____ | _____ | _____ | 128 |
| World Health Organization[11] | 2015 | Somalia | Somalia | 2012 | Globo can | 2012 | 40.5 | _____ | _____ | _____ | 1260 |
| Y. Bhurgri,[29] | 2004 | Pakistan | Karachi | 1995-2002 | Cancer Registry | 2000 | 69.1 | _____ | _____ | _____ |  |
| Y. Bhurgri, [30] | 2002 | Pakistan | Karachi | 1999 | Cancer Registry | 1999 | 40.7 | _____ | _____ | _____ | 1094 |
| Y. Bhurgri, [30] | 2002 | Pakistan | Quetta | 1999 | Cancer Registry | 1999 | 11.8 | _____ | _____ | _____ | 43 |
| Z. Almasi,[87] | 2016 | Iran | Iran | 2012 | Globo can | 2008 | 24.3 | _____ | 14.4 | _____ | _____ |
| Z. Bodalal, | 2014 | Libya | Benghazi | 2002 | Globo can | 2002 | 17.4 | _____ | _____ | _____ | 210 |
| Z. Bodalal, | 2014 | Libya | Benghazi | 2003 | Oncology | 2003 | 22.9 | _____ | _____ | _____ | 210 |
| Z. Bodalal, | 2014 | Libya | Western Libya | 2007 | Oncology | 2007 | 31.1 | _____ | _____ | _____ | 210 |
| Z. Bodalal, | 2014 | Libya | Benghazi | 2012 | Oncology | 2012 | 37.4 | _____ | _____ | _____ | 210 |
| Z. Bouchbika,[31] | 2014 | Tunisia | North | 2004-2006 | Cancer Registry | 2005 | 31.8 | _____ | _____ | _____ | _____ |
| Z. Bouchbika,[31] | 2014 | Morocco | Casablanca | 2005-2007 | Cancer Registry | 2006 | 36.4 | _____ | 37.5 | _____ | 2119 |
| J. Ferlay,[43] | 2014 | Qatar | Qatar | 2003-2007 | IARC | 2005 | 45.7 | _____ | _____ | _____ | 150 |
| J. Ferlay,[43] | 2014 | Iran | Golestan | 2005-2007 | IARC | 2006 | 28 | _____ | _____ | _____ | 558 |
| J. Ferlay,[43] | 2014 | Bahrain | Bahrain | 2005-2007 | IARC | 2006 | 56 | _____ | _____ | _____ | 460 |
| S.El-Zaemey,[36] | 2012 | Yemen | Yemen | 2008 | Globo can | 2008 | 20.8 | _____ | _____ | _____ | _____ |
| S.El-Zaemey,[36] | 2012 | Saudi Arabia | Saudi Arabia | 2008 | Globo can | 2008 | 22.4 | _____ | _____ | _____ | _____ |
| S.El-Zaemey,[36] | 2012 | Syria | Syria | 2008 | Globo can | 2008 | 23 | _____ | _____ | _____ | _____ |
| S.El-Zaemey,[36] | 2012 | Libya | Libya | 2008 | Globo can | 2008 | 23.3 | _____ | _____ | _____ | _____ |
| S.El-Zaemey,[36] | 2012 | Oman | Oman | 2008 | Globo can | 2008 | 28.3 | _____ | _____ | _____ | _____ |
| S.El-Zaemey,[36] | 2012 | Iraq | Iraq | 2008 | Globo can | 2008 | 31.1 | _____ | _____ | _____ | _____ |
| S.El-Zaemey,[36] | 2012 | UAE | UAE | 2008 | Globo can | 2008 | 36.7 | _____ | _____ | _____ | _____ |
| S.El-Zaemey,[36] | 2012 | Egypt | Egypt | 2008 | Globo can | 2008 | 37.3 | _____ | _____ | _____ | _____ |
| S.El-Zaemey,[36] | 2012 | Qatar | Qatar | 2008 | Globo can | 2008 | 38.1 | _____ | _____ | _____ | _____ |
| S.El-Zaemey,[36] | 2012 | Jordan | Jordan | 2008 | Globo can | 2008 | 47 | _____ | _____ | _____ | _____ |
| S.El-Zaemey,[36] | 2012 | Kuwait | Kuwait | 2008 | Globo can | 2008 | 47.7 | _____ | _____ | _____ | _____ |
| S.El-Zaemey,[36] | 2012 | Lebanon | Lebanon | 2008 | Globo can | 2008 | 55.4 | _____ | _____ | _____ | _____ |
| R. R. Hamadeh,[76] | 2014 | Bahrain | Bahrain | 2008 | Globo can | 2008 | 49.8 | _____ | _____ | _____ | _____ |
| R. R. Hamadeh,[76] | 2014 | Tunisia | Tunisia | 2008 | Globo can | 2008 | 30.3 | _____ | _____ | _____ | _____ |

| Author | **Year of publish Data** | **Country** | **City** | **Duration of study** | **Source of Data** | **Year of Data** | **ASR** | **SE ASR** | **ICR** | **SE ICR** | **Total Breast cases** |
| --- | --- | --- | --- | --- | --- | --- | --- | --- | --- | --- | --- |
| Fitzmaurice,[1] | 2017 | Qatar | Qatar | 2005-2015 | Cancer Registry | 2005 | 62.9 | ____ | ____ | ____ | 105 |
| Fitzmaurice,[1] | 2017 | Bahrain | Bahrain | 2005-2015 | Cancer Registry | 2005 | 66.6 | ____ | ____ | ____ | 154 |
| Fitzmaurice,[1] | 2017 | Djibouti | Djibouti | 2005-2015 | Cancer Registry | 2005 | 55.6 | ____ | ____ | ____ | 120 |
| Fitzmaurice,[1] | 2017 | Libya | Libya | 2005-2015 | Cancer Registry | 2005 | 33.7 | ____ | ____ | ____ | 585 |
| Fitzmaurice,[1] | 2017 | Oman | Oman | 2005-2015 | Cancer Registry | 2005 | 28.9 | ____ | ____ | ____ | 149 |
| Fitzmaurice,[1] | 2017 | Kuwait | Kuwait | 2005-2015 | Cancer Registry | 2005 | 58.6 | ____ | ____ | ____ | 293 |
| Fitzmaurice,[1] | 2017 | UAE | UAE | 2005-2015 | Cancer Registry | 2005 | 68.7 | ____ | ____ | ____ | 449 |
| Fitzmaurice,[1] | 2017 | Saudi Arabia | Saudi Arabia | 2005-2015 | Cancer Registry | 2005 | 27.5 | ____ | ____ | ____ | 1550 |
| Fitzmaurice,[1] | 2017 | Lebanon | Lebanon | 2005-2015 | Cancer Registry | 2005 | 84.2 | ____ | ____ | ____ | 1328 |
| Fitzmaurice,[1] | 2017 | Morocco | Morocco | 2005-2015 | Cancer Registry | 2005 | 68.7 | ____ | ____ | ____ | 8084 |
| Fitzmaurice,[1] | 2017 | Jordan | Jordan | 2005-2015 | Cancer Registry | 2005 | 65 | ____ | ____ | ____ | 883 |
| Fitzmaurice,[1] | 2017 | Somalia | Somalia | 2005-2015 | Cancer Registry | 2005 | 51.2 | ____ | ____ | ____ | 1062 |
| Fitzmaurice,[1] | 2017 | Tunisia | Tunisia | 2005-2015 | Cancer Registry | 2005 | 57.4 | ____ | ____ | ____ | 2506 |
| Fitzmaurice,[1] | 2017 | Syria | Syria | 2005-2015 | Cancer Registry | 2005 | 31.6 | ____ | ____ | ____ | 1405 |
| Fitzmaurice,[1] | 2017 | Yemen | Yemen | 2005-2015 | Cancer Registry | 2005 | 70.1 | ____ | ____ | ____ | 3440 |
| Fitzmaurice,[1] | 2017 | Afghanistan | Afghanistan | 2005-2015 | Cancer Registry | 2005 | 113.5 | ____ | ____ | ____ | 6340 |
| Fitzmaurice,[1] | 2017 | Sudan | Sudan | 2005-2015 | Cancer Registry | 2005 | 55 | ____ | ____ | ____ | 4533 |
| Fitzmaurice,[1] | 2017 | Iraq | Iraq | 2005-2015 | Cancer Registry | 2005 | 117.5 | ____ | ____ | ____ | 8425 |
| Fitzmaurice,[1] | 2017 | Pakistan | Pakistan | 2005-2015 | Cancer Registry | 2005 | 118.7 | ____ | ____ | ____ | 50392 |
| Fitzmaurice,[1] | 2017 | Egypt | Egypt | 2005-2015 | Cancer Registry | 2005 | 49.2 | ____ | ____ | ____ | 13122 |
| C. Fitzmaurice, [93] | 2015 | Djibouti | Djibouti | 1990-2013 | Cancer Registry | 2013 | 51.66 | ____ | ____ | ____ | 120 |
| C. Fitzmaurice, [93] | 2015 | Bahrain | Bahrain | 1990-2013 | Cancer Registry | 2013 | 61.48 | ____ | ____ | ____ | 240 |
| C. Fitzmaurice, [93] | 2015 | Qatar | Qatar | 1990-2013 | Cancer Registry | 2013 | 54.7 | ____ | ____ | ____ | 170 |
| C. Fitzmaurice, [93] | 2015 | Kuwait | Kuwait | 1990-2013 | Cancer Registry | 2013 | 57.83 | ____ | ____ | ____ | 440 |
| C. Fitzmaurice, [93] | 2015 | UAE | UAE | 1990-2013 | Cancer Registry | 2013 | 52.94 | ____ | ____ | ____ | 750 |
| C. Fitzmaurice, [93] | 2015 | Lebanon | Lebanon | 1990-2013 | Cancer Registry | 2013 | 82.88 | ____ | ____ | ____ | 1760 |
| C. Fitzmaurice, [93] | 2015 | Jordan | Jordan | 1990-2013 | Cancer Registry | 2013 | 44.24 | ____ | ____ | ____ | 990 |
| C. Fitzmaurice, [93] | 2015 | Libya | Libya | 1990-2013 | Cancer Registry | 2013 | 34.43 | ____ | ____ | ____ | 850 |
| C. Fitzmaurice, [93] | 2015 | Tunisia | Tunisia | 1990-2013 | Cancer Registry | 2013 | 39.2 | ____ | ____ | ____ | 2160 |
| MP. Curado,[32] | 2007 | Oman | Oman | 1998-2001 | IARC | 2000 | 14.6 | ____ | ____ | ____ | 265 |
| MP. Curado,[32] | 2007 | Pakistan | Karachi South | 1998-2002 | IARC | 2000 | 69 | ____ | ____ | ____ | 1497 |
| MP. Curado,[32] | 2007 | Tunisia | Sousee | 1998-2002 | IARC | 2000 | 29.8 | ____ | ____ | ____ | 308 |
| MP. Curado,[32] | 2007 | Egypt | Gharbiah | 1998-2002 | IARC | 2000 | 42.5 |  |  |  | 4226 |
| MP. Curado,[32] | 2007 | Kuwait | Kuwait | 1998-2002 | IARC | 2000 | 41.3 |  |  |  | 551 |

| **Author** | **Year of publish Data** | **Country** | **City** | **Duration of study** | **Source of Data** | **Year of Data** | **ASR** | **SE ASR** | **ICR** | **SE ICR** | **Total Breast cases** |
| --- | --- | --- | --- | --- | --- | --- | --- | --- | --- | --- | --- |
| C. Fitzmaurice, [93] | 2015 | Somalia | Somalia | 1990-2013 | Cancer Registry | 2013 | 29.46 | ____ | ____ | ____ | 770 |
| C. Fitzmaurice, [93] | 2015 | Syria | Syria | 1990-2013 | Cancer Registry | 2013 | 10.9 | ____ | ____ | ____ | 730 |
| C. Fitzmaurice, [93] | 2015 | Yemen | Yemen | 1990-2013 | Cancer Registry | 2013 | 32.12 | ____ | ____ | ____ | 2050 |
| C. Fitzmaurice, [93] | 2015 | Oman | Oman | 1990-2013 | Cancer Registry | 2013 | 29.9 | ____ | ____ | ____ | 230 |
| C. Fitzmaurice, [93] | 2015 | Saudi Arabia | Saudi Arabia | 1990-2013 | Cancer Registry | 2013 | 20.9 | ____ | ____ | ____ | 1540 |
| C. Fitzmaurice, [93] | 2015 | Afghanistan | Afghanistan | 1990-2013 | Cancer Registry | 2013 | 34.1 | ____ | ____ | ____ | 2500 |
| C. Fitzmaurice, [93] | 2015 | Morocco | Morocco | 1990-2013 | Cancer Registry | 2013 | 42.48 | ____ | ____ | ____ | 6070 |
| C. Fitzmaurice, [93] | 2015 | Iraq | Iraq | 1990-2013 | Cancer Registry | 2013 | 55.71 | ____ | ____ | ____ | 5490 |
| C. Fitzmaurice, [93] | 2015 | Sudan | Sudan | 1990-2013 | Cancer Registry | 2013 | 36.47 | ____ | ____ | ____ | 3840 |
| C. Fitzmaurice, [93] | 2015 | Egypt | Egypt | 1990-2013 | Cancer Registry | 2013 | 37.13 | ____ | ____ | ____ | 12150 |
| C. Fitzmaurice, [93] | 2015 | Pakistan | Pakistan | 1990-2013 | Cancer Registry | 2013 | 93.4 | ____ | ____ | ____ | 54430 |
| C. Fitzmaurice,[1] | 2017 | Djibouti | Djibouti | 2005-2015 | Cancer Registry | 2015 | 69.7 | ____ | ____ | ____ | 200 |
| C. Fitzmaurice,[1] | 2017 | Bahrain | Bahrain | 2005-2015 | Cancer Registry | 2015 | 68.3 | ____ | ____ | ____ | 278 |
| C. Fitzmaurice,[1] | 2017 | Qatar | Qatar | 2005-2015 | Cancer Registry | 2015 | 71 | ____ | ____ | ____ | 280 |
| C. Fitzmaurice,[1] | 2017 | Yemen | Yemen | 2005-2015 | Cancer Registry | 2015 | 87.1 | ____ | ____ | ____ | 5800 |
| C. Fitzmaurice,[1] | 2017 | Oman | Oman | 2005-2015 | Cancer Registry | 2015 | 35.5 | ____ | ____ | ____ | 303 |
| C. Fitzmaurice,[1] | 2017 | Kuwait | Kuwait | 2005-2015 | Cancer Registry | 2015 | 73 | ____ | ____ | ____ | 642 |
| C. Fitzmaurice,[1] | 2017 | UAE | UAE | 2005-2015 | Cancer Registry | 2015 | 82.4 | ____ | ____ | ____ | 1298 |
| C. Fitzmaurice,[1] | 2017 | Lebanon | Lebanon | 2005-2015 | Cancer Registry | 2015 | 89.5 | ____ | ____ | ____ | 2214 |
| C. Fitzmaurice,[1] | 2017 | Libya | Libya | 2005-2015 | Cancer Registry | 2015 | 40.1 | ____ | ____ | ____ | 975 |
| C. Fitzmaurice,[1] | 2017 | Jordan | Jordan | 2005-2015 | Cancer Registry | 2015 | 58.2 | ____ | ____ | ____ | 1305 |
| C. Fitzmaurice,[1] | 2017 | Tunisia | Tunisia | 2005-2015 | Cancer Registry | 2015 | 65.6 | ____ | ____ | ____ | 3786 |
| C. Fitzmaurice,[1] | 2017 | Somalia | Somalia | 2005-2015 | Cancer Registry | 2015 | 65.8 | ____ | ____ | ____ | 1659 |
| C. Fitzmaurice,[1] | 2017 | Syria | Syria | 2005-2015 | Cancer Registry | 2015 | 39.8 | ____ | ____ | ____ | 2326 |
| C. Fitzmaurice,[1] | 2017 | Saudi Arabia | Saudi Arabia | 2005-2015 | Cancer Registry | 2015 | 30.9 | ____ | ____ | ____ | 2584 |
| C. Fitzmaurice,[1] | 2017 | Afghanistan | Afghanistan | 2005-2015 | Cancer Registry | 2015 | 147.9 | ____ | ____ | ____ | 1138 |
| C. Fitzmaurice,[1] | 2017 | Morocco | Morocco | 2005-2015 | Cancer Registry | 2015 | 82.9 | ____ | ____ | ____ | 12855 |
| C. Fitzmaurice,[1] | 2017 | Iraq | Iraq | 2005-2015 | Cancer Registry | 2015 | 123 | ____ | ____ | ____ | 12734 |
| C. Fitzmaurice,[1] | 2017 | Sudan | Sudan | 2005-2015 | Cancer Registry | 2015 | 67.7 | ____ | ____ | ____ | 7509 |
| C. Fitzmaurice,[1] | 2017 | Egypt | Egypt | 2005-2015 | Cancer Registry | 2015 | 56.2 | ____ | ____ | ____ | 19192 |
| C. Fitzmaurice,[1] | 2017 | Pakistan | Pakistan | 2005-2015 | Cancer Registry | 2015 | 135.8 | ____ | ____ | ____ | 77329 |

| **Author** | **Year of publish Data** | **Country** | **City** | **Duration of study** | **Source of Data** | **Year of Data** | **ASR** | **SE ASR** | **ICR** | **SE ICR** | Total Breast cases |
| --- | --- | --- | --- | --- | --- | --- | --- | --- | --- | --- | --- |
| Roshandel; Gholam Reza, [82] | 2018 | Iran | Tehran | 2015-2016 | Cancer Registry | 2014 | 51 | ____ | ____ | ____ | 3503 |
| Roshandel; Gholam Reza, [82] | 2018 | Iran | Ardabil | 2015-2016 | Cancer Registry | 2014 | 12.7 | ____ | ____ | ____ | 79 |
| Roshandel; Gholam Reza, [82] | 2018 | Iran | Kerman | 2015-2016 | Cancer Registry | 2014 | 29.9 | ____ | ____ | ____ | 406 |
| Roshandel; Gholam Reza, [82] | 2018 | Iran | Kermanshah | 2015-2016 | Cancer Registry | 2014 | 28.1 | ____ | ____ | ____ | 288 |
| Roshandel; Gholam Reza, [82] | 2018 | Iran | Alborz | 2015-2016 | Cancer Registry | 2014 | 31.6 | ____ | ____ | ____ | 410 |
| Roshandel; Gholam Reza, [82] | 2018 | Iran | Kordestan | 2015-2016 | Cancer Registry | 2014 | 15.1 | ____ | ____ | ____ | 111 |
| Roshandel; Gholam Reza, [82] | 2018 | Iran | Kohkilie boir ahmad | 2015-2016 | Cancer Registry | 2014 | 21.8 | ____ | ____ | ____ | 67 |
| Roshandel; Gholam Reza, [82] | 2018 | Iran | Khorasan jonobi | 2015-2016 | Cancer Registry | 2014 | 22.9 | ____ | ____ | ____ | 83 |
| Roshandel; Gholam Reza, [82] | 2018 | Iran | Golestan | 2015-2016 | Cancer Registry | 2014 | 33.1 | ____ | ____ | ____ | 308 |
| Roshandel; Gholam Reza, [82] | 2018 | Iran | Gilan | 2015-2016 | Cancer Registry | 2014 | 26.3 | ____ | ____ | ____ | 421 |
| Roshandel; Gholam Reza, [82] | 2018 | Iran | Golestan | 2015-2016 | Cancer Registry | 2014 | 33.1 | ____ | ____ | ____ | 308 |
| Roshandel; Gholam Reza, [82] | 2018 | Iran | Mazandran | 2015-2016 | Cancer Registry | 2014 | 31.9 | ____ | ____ | ____ | 585 |
| Roshandel; Gholam Reza, [82] | 2018 | Iran | Fars | 2015-2016 | Cancer Registry | 2014 | 35.8 | ____ | ____ | ____ | 836 |
| Roshandel; Gholam Reza, [82] | 2018 | Iran | Semnan | 2015-2016 | Cancer Registry | 2014 | 46.9 | ____ | ____ | ____ | 157 |
| Roshandel; Gholam Reza, [82] | 2018 | Iran | Isfahan | 2015-2016 | Cancer Registry | 2014 | 42.7 | ____ | ____ | ____ | 1131 |
| Roshandel; Gholam Reza, [82] | 2018 | Iran | Azarbaijan gharbi | 2015-2016 | Cancer Registry | 2014 | 24.8 | ____ | ____ | ____ | 380 |
| Roshandel; Gholam Reza, [82] | 2018 | Iran | Azarbaijan sharghi | 2015-2016 | Cancer Registry | 2014 | 23.7 | ____ | ____ | ____ | 491 |
| Roshandel; Gholam Reza, [82] | 2018 | Iran | Booshehr | 2015-2016 | Cancer Registry | 2014 | 32 | ____ | ____ | ____ | 140 |
| Roshandel; Gholam Reza, [82] | 2018 | Iran | Chaharmahal bakhtiari | 2015-2016 | Cancer Registry | 2014 | 23.4 | ____ | ____ | ____ | 104 |
| Roshandel; Gholam Reza, [82] | 2018 | Iran | Ghazvin | 2015-2016 | Cancer Registry | 2014 | 23.1 | ____ | ____ | ____ | 144 |
| Roshandel; Gholam Reza, [82] | 2018 | Iran | Hamadan | 2015-2016 | Cancer Registry | 2014 | 19.6 | ____ | ____ | ____ | 182 |
| Roshandel; Gholam Reza, [82] | 2018 | Iran | Hormozgan | 2015-2016 | Cancer Registry | 2014 | 26.4 | ____ | ____ | ____ | 175 |
| Roshandel; Gholam Reza, [82] | 2018 | Iran | Ilam | 2015-2016 | Cancer Registry | 2014 | 31.1 | ____ | ____ | ____ | 82 |
| Roshandel; Gholam Reza, [82] | 2018 | Iran | Khorasan rezavi | 2015-2016 | Cancer Registry | 2014 | 45.9 | ____ | ____ | ____ | 1373 |
| Roshandel; Gholam Reza, [82] | 2018 | Iran | Khozestan | 2015-2016 | Cancer Registry | 2014 | 38.4 | ____ | ____ | ____ | 778 |
| Roshandel; Gholam Reza, [82] | 2018 | Iran | Lorestan | 2015-2016 | Cancer Registry | 2014 | 14.7 | ____ | ____ | ____ | 132 |
| Roshandel; Gholam Reza, [82] | 2018 | Iran | Markazi | 2015-2016 | Cancer Registry | 2014 | 21.8 | ____ | ____ | ____ | 164 |
| Roshandel; Gholam Reza, [82] | 2018 | Iran | Sistan and Baluchestan | 2015-2016 | Cancer Registry | 2014 | 16.4 | ____ | ____ | ____ | 147 |
| Roshandel; Gholam Reza, [82] | 2018 | Iran | Yazd | 2015-2016 | Cancer Registry | 2014 | 47.5 | ____ | ____ | ____ | 229 |
| Roshandel; Gholam Reza, [82] | 2018 | Iran | zanjan | 2015-2016 | Cancer Registry | 2014 | 22.2 | ____ | ____ | ____ | 117 |

| Author | Year of publish Data | Country | City | Duration of study | Source of Data | Year of Data | ASR | SE ASR | ICR | SE ICR | Total Breast cases |
| --- | --- | --- | --- | --- | --- | --- | --- | --- | --- | --- | --- |
| Rajesh Sharma[85] | 2018 | Afghanistan | Afghanistan | 2016 | Globo can | 2016 | 34.38 | ____ | ____ | ____ | 2735 |
| Rajesh Sharma[85] | 2018 | sudan | sudan | 2016 | Globo can | 2016 | 29.58 | ____ | ____ | ____ | 3537 |
| Rajesh Sharma[85] | 2018 | syria | syria | 2016 | Globo can | 2016 | 17.12 | ____ | ____ | ____ | 1046 |
| Rajesh Sharma[85] | 2018 | Tunisia | Tunisia | 2016 | Globo can | 2016 | 41.75 | ____ | ____ | ____ | 2504 |
| Rajesh Sharma[85] | 2018 | Yemen | Yemen | 2016 | Globo can | 2016 | 26.63 | ____ | ____ | ____ | 1855 |
| Rajesh Sharma[85] | 2018 | Egypt | Egypt | 2016 | Globo can | 2016 | 23.65 | ____ | ____ | ____ | 8427 |
| Rajesh Sharma[85] | 2018 | Iraq | Iraq | 2016 | Globo can | 2016 | 35.89 | ____ | ____ | ____ | 3785 |
| Rajesh Sharma[85] | 2018 | Morroco | Morroco | 2016 | Globo can | 2016 | 44.58 | ____ | ____ | ____ | 7558 |
| Rajesh Sharma[85] | 2018 | Pakistan | Pakistan | 2016 | Globo can | 2016 | 42.51 | ____ | ____ | ____ | 26103 |
| Rajesh Sharma[85] | 2018 | Iran | Iran | 2016 | Globo can | 2016 | 31.3 | ____ | ____ | ____ | 11042 |
| Rajesh Sharma[85] | 2018 | Jordan | Jordan | 2016 | Globo can | 2016 | 49.88 | ____ | ____ | ____ | 1216 |
| Rajesh Sharma[85] | 2018 | Lebanon | Lebanon | 2016 | Globo can | 2016 | 94.53 | ____ | ____ | ____ | 2451 |
| Rajesh Sharma[85] | 2018 | Libya | Libya | 2016 | Globo can | 2016 | 41.5 | ____ | ____ | ____ | 1127 |
| Rajesh Sharma[85] | 2018 | Saudi Arabia | Saudi Arabia | 2016 | Globo can | 2016 | 36.43 | ____ | ____ | ____ | 3351 |
| Abdolreza Faze[83] | 2019 | Iran | Golestan | 2004 | cancer registry | 2004 | 18.3 | ____ | ____ | ____ | 111 |
| Abdolreza Faze[83] | 2019 | Iran | Golestan | 2005 | cancer registry | 2005 | 25.2 | ____ | ____ | ____ | 153 |
| Abdolreza Faze[83] | 2019 | Iran | Golestan | 2006 | cancer registry | 2006 | 27.5 | ____ | ____ | ____ | 183 |
| Abdolreza Faze[83] | 2019 | Iran | Golestan | 2007 | cancer registry | 2007 | 31.8 | ____ | ____ | ____ | 224 |
| Abdolreza Faze[83] | 2019 | Iran | Golestan | 2008 | cancer registry | 2008 | 304 | ____ | ____ | ____ | 215 |
| Abdolreza Faze[83] | 2019 | Iran | Golestan | 2009 | cancer registry | 2009 | 29.7 | ____ | ____ | ____ | 213 |
| Abdolreza Faze[83] | 2019 | Iran | Golestan | 2010 | cancer registry | 2010 | 31 | ____ | ____ | ____ | 225 |
| Abdolreza Faze[83] | 2019 | Iran | Golestan | 2011 | cancer registry | 2011 | 28.9 | ____ | ____ | ____ | 234 |
| Abdolreza Faze[83] | 2019 | Iran | Golestan | 2012 | cancer registry | 2012 | 33.7 | ____ | ____ | ____ | 263 |
| Abdolreza Faze[83] | 2019 | Iran | Golestan | 2013 | cancer registry | 2013 | 31.7 | ____ | ____ | ____ | 285 |
| World Health Organization[86] | 2019 | pakistan | pakistan | 2018 | Globo can | 2018 | 43.9 | ____ | ____ | ____ | 34066 |
| World Health Organization[86] | 2019 | Afghanistan | Afghanistan | 2018 | Globo can | 2018 | 30 | ____ | ____ | ____ | 3062 |
| World Health Organization[86] | 2019 | Bahrain | Bahrain | 2018 | Globo can | 2018 | 44.1 | ____ | ____ | ____ | 227 |
| World Health Organization[86] | 2019 | Iran | Iran | 2018 | Globo can | 2018 | 31 | ____ | ____ | ____ | 13776 |
| World Health Organization[86] | 2019 | Iraq | Iraq | 2018 | Globo can | 2018 | 38.4 | ____ | ____ | ____ | 5141 |
| World Health Organization[86] | 2019 | Jordan | Jordan | 2018 | Globo can | 2018 | 57.4 | ____ | ____ | ____ | 2143 |

| Author | Year of publish Data | Country | City | Duration of study | Source of Data | Year of Data | ASR | SE ASR | ICR | SE ICR | Total Breast cases |
| --- | --- | --- | --- | --- | --- | --- | --- | --- | --- | --- | --- |
| World Health Organization[86] | 2019 | Lebanon | Lebanon | 2018 | Globo can | 2018 | 97.6 | ____ | ____ | ____ | 3219 |
| World Health Organization[86] | 2019 | oman | oman | 2018 | Globo can | 2018 | 34.7 | ____ | ____ | ____ | 454 |
| World Health Organization[86] | 2019 | pakistan | pakistan | 2018 | Globo can | 2018 | 43.9 | ____ | ____ | ____ | 34066 |
| World Health Organization[86] | 2019 | Qatar | Qatar | 2018 | Globo can | 2018 | 42.1 | ____ | ____ | ____ | 190 |
| World Health Organization[86] | 2019 | Saudi Arabia | Saudi Arabia | 2018 | Globo can | 2018 | 27.3 | ____ | ____ | ____ | 3629 |
| World Health Organization[86] | 2019 | syria | syria | 2018 | Globo can | 2018 | 67.3 | ____ | ____ | ____ | 4935 |
| World Health Organization[86] | 2019 | united arab | united arab | 2018 | Globo can | 2018 | 52.9 | ____ | ____ | ____ | 1054 |
| World Health Organization[86] | 2019 | Yemen | Yemen | 2018 | Globo can | 2018 | 24.9 | ____ | ____ | ____ | 2445 |
| World Health Organization[86] | 2019 | egypt | egypt | 2018 | Globo can | 2018 | 52.2 | ____ | ____ | ____ | 25399 |
| World Health Organization[86] | 2019 | Libya | Libya | 2018 | Globo can | 2018 | 23.2 | ____ | ____ | ____ | 753 |
| World Health Organization[86] | 2019 | morroco | morroco | 2018 | Globo can | 2018 | 51 | ____ | ____ | ____ | 10136 |
| World Health Organization[86] | 2019 | sudan | sudan | 2018 | Globo can | 2018 | 39.2 | ____ | ____ | ____ | 5677 |
| World Health Organization[86] | 2019 | tunisia | tunisia | 2018 | Globo can | 2018 | 32.2 | ____ | ____ | ____ | 2305 |
| World Health Organization[86] | 2019 | djibouti | djibouti | 2018 | Globo can | 2018 | 41.2 | ____ | ____ | ____ | 176 |
| Roshandel; Gholam Reza[84] | 2019 | Iran | Golestan | 2014 | cancer registry | 2014 | 34.5 | ____ | ____ | ____ | 13120 |
|  |  |  |  |  |  |  |  |  |  |  |  |
|  |  |  |  |  |  |  |  |  |  |  |  |
|  |  |  |  |  |  |  |  |  |  |  |  |
|  |  |  |  |  |  |  |  |  |  |  |  |
|  |  |  |  |  |  |  |  |  |  |  |  |
|  |  |  |  |  |  |  |  |  |  |  |  |
|  |  |  |  |  |  |  |  |  |  |  |  |
|  |  |  |  |  |  |  |  |  |  |  |  |
|  |  |  |  |  |  |  |  |  |  |  |  |
|  |  |  |  |  |  |  |  |  |  |  |  |
|  |  |  |  |  |  |  |  |  |  |  |  |
|  |  |  |  |  |  |  |  |  |  |  |  |
|  |  |  |  |  |  |  |  |  |  |  |  |
|  |  |  |  |  |  |  |  |  |  |  |  |
|  |  |  |  |  |  |  |  |  |  |  |  |

**Graph S1: The ASR trend of breast cancer in Eastern Mediterian Region by qulity of data registration throughout 1998-2019**

**GraphS2: The ASR trend of breast cancer in Eastern Mediterian Region by qulity of data registration throughout 1998-2019**

1:Group1 (High Quality of Data), 2:Group2 (Medium Quality of Data), 3:Group3 (Low Quality of Data)

**FigureS1: Geographic Distribution of ASR of breast cancer in Eastern Mediterranean Countries from 1998 to 2018**
